# Transcriptome analysis of smooth cordgrass (*Spartina alterniflora* Loisel), a monocot halophyte, reveals candidate genes involved in its adaptation to salinity

**DOI:** 10.1186/s12864-016-3017-3

**Published:** 2016-08-19

**Authors:** Renesh Bedre, Venkata Ramanarao Mangu, Subodh Srivastava, Luis Eduardo Sanchez, Niranjan Baisakh

**Affiliations:** 1 School of Plant, Environmental and Soil Sciences, Louisiana State University Agricultural Center, Baton Rouge, LA 70803 USA; 2 Department of Genetics and Biochemistry, Clemson University, Clemson, SC 29634 USA; 3 Current address: Centro de Investigaciones Biotecnológicas del Ecuador (CIBE), Km 30.5 Via Perimetral, Guayaquil, Ecuador

**Keywords:** Gene expression, GS-FLX, Salinity, *Spartina alterniflora*, Transcriptome

## Abstract

**Background:**

Soil salinity affects growth and yield of crop plants. Plants respond to salinity by physiological and biochemical adjustments through a coordinated regulation and expression of a cascade of genes. Recently, halophytes have attracted attention of the biologists to understand their salt adaptation mechanisms. *Spartina alterniflora* (smooth cordgrass) is a Louisiana native monocot halophyte that can withstand salinity up to double the strength of sea water. To dissect the molecular mechanisms underlying its salinity adaptation, leaf and root transcriptome of *S. alterniflora* was sequenced using 454/GS-FLX.

**Results:**

Altogether, 770,690 high quality reads with an average length 324-bp were assembled de novo into 73,131 contigs (average 577-bp long) with 5.9X sequence coverage. Most unigenes (95 %) annotated to proteins with known functions, and had more than 90 % similarity to rice genes. About 28 % unigenes were considered specific to *S. alterniflora*. Digital expression profiles revealed significant enrichment (*P* < 0.01) of transporters, vacuolar proton pump members and transcription factors under salt stress, which suggested the role of ion homeostasis and transcriptional regulation in the salinity adaptation of this grass. Also, 10,805 SSRs markers from 9457 unigenes were generated and validated through genetic diversity analysis among 13 accessions of *S. alterniflora*.

**Conclusions:**

The present study explores the transcriptome of *S. alterniflora* to understand the gene regulation under salt stress in halophytes. The sequenced transcriptome (control and salt-regulated) of *S. alterniflora* provides a platform for further gene finding studies in grasses. This study and our previously published studies suggested that *S. alterniflora* is a rich reservoir of salt tolerance genes that can be used to develop salt tolerant cereal crops, especially rice, a major food crop of global importance.

**Electronic supplementary material:**

The online version of this article (doi:10.1186/s12864-016-3017-3) contains supplementary material, which is available to authorized users.

## Background

Soil salinity negatively affects growth and development of plants by impairing the physiological and biochemical processes, thereby reducing crop yield and quality, or in severe case, death of the plant [1–3]. Salinity causes constant reduction in arable lands [4], and rise in the sea water level due to inevitable global climate change will only worsen this situation in future. This is further complicated by the increasing salinity of irrigation water in dry areas, perpetual hurricanes that result in inundation of sea water into cultivated land, etc. Future agricultural production will rely on our ability to develop and grow salt tolerant food and fiber crops in salt-affected areas [5]. Most crop plants are sensitive to salt stress and the extent of damage varies with the species, genotypes within a species, phenology of the plant, and the severity of soil salinity. Conventional breeding has exploited the understanding of the genetic basis of salt tolerance and the genotypic variations within primary and secondary gene pools to develop salt tolerant crops. However, the progress to this end has been slow and little due to the complexity and multigenic inheritance with low to moderate heritability of salt tolerance traits [6]. Therefore, systems level understanding of plant biology, physiology, genetics and biochemistry of salt stress responses is required to translate the knowledge to designing salt tolerant crops. Substantial progress has been made in the past in the understanding and identification of different salt tolerance mechanisms in the model glycophyte *Arabidopsis thaliana*. However, translation of *Arabidopsis* resources to more distant crops, such as rice, has been limited due to significant differences in their signal transduction and gene regulatory networks [7].

Halophytes have the ability to complete their life cycle at a salt concentration of at least 200 mM NaCl or up to 5 g/l of total dissolved salt in irrigation water [8, 9], whereas most sensitive crop plants, such as rice, can be severely affected at as low as 20 to 50 mM NaCl [10]. A true halophyte remains viable at or beyond sea water salinity [11, 12]. Optimal growth of halophytes is observed at soil salinity between 200 to 400 mM NaCl [13]. Several mechanisms are known to be operational at the cellular, organizational, and whole plant level in halophytes for their adaptation to soil salinity [14]. Genetically, high tolerance to salinity in halophytes may be due to (1) superior gene regulatory mechanism, (2) superior alleles of salt-responsive genes, and (3) presence of unique/novel genes or processes [15]. Importantly, halophytes have comparative advantages over glycophytes in their ability to determine the nature of transporters involved in the uptake toxic Na^+^ [16], accumulation of high concentrations of K^+^ [12], and preferential accumulation of salt (up to 50 % of shoot dry weight) to balance osmotic potential [9]. Comparative expression profiling studies of *Thellungiella halophila* with *A. thaliana* suggested that differences in the regulatory networks involved in stress perception and subsequent triggering of stress-responsive genes in halophytes may account for their stress anticipatory preparedness and superior adaptation responses [15, 17–19].

Previous studies on halophytes have mostly been focused on dicot halophytes, such as *T. halophila* due to its close similarity with *A. thaliana*. Transfer of such knowledge to major cereal food crops like rice has been limited due to differences in developmental and anatomical features associated with salt tolerance mechanisms of dicot and monocot halophytes. For example, dicots, being succulent, accumulate more Na^+^ in their shoots than monocot halophytes [20]. Therefore, an effort to develop and exploit molecular genetic resources of wild relatives within cereal species is necessary to identify loci involved in environmental adaptation and their subsequent translation to improve salinity tolerance of cereals [21]. But, wild relatives of a crop (e.g., rice), which has long term adaptability to salinity, may not always exist. This warrants for elucidation of regulation of adaptive processes of a grass halophyte involved in response to high and prolonged salinity [12].

Among different monocotyledonous halophytes, *Spartina alterniflora* (smooth cordgrass), a dominant salt marsh grass along the Atlantic and gulf coasts of the U.S., has exceptionally high tolerance to salinity, and is known to possess all possible mechanisms of salt tolerance, such as ion exclusion at root level and ion secretion in leaves through salt glands [22], vacuolar sequestration of toxic Na^+^ [23], maintenance of reduced osmotic potential through synthesis and accumulation of compatible solutes [24] and maintenance of photosynthesis [25]. *S. alterniflora* is an aneu-hexaploid (2n = 6x = 62) belonging to the subfamily Chloridoideae of family Poaceae [26]. Flow cytometry analysis showed that among *Spartina* species, *S. alterniflora* has the largest genome (1763.9 Mbp), more than four times of the size of the rice genome [27]. Previous studies with small-scale transcriptome analysis showed that *S. alterniflora* showed 80–90 % similarity to rice at the DNA and protein sequences level [28, 29]. Furthermore, *Agrobacterium*-mediated introgression of *S. alterniflora* genes, such as vacuolar H^+^-ATPase subunit c1 (*SaVHAc1*), adenosine diphosphate ribosylation factor (*SaARF1*) and myo-inositol phosphate synthase (*SaINO1*), into rice, *Arabidopsis* and tobacco has demonstrated abiotic stress tolerance of transgenic overexpressers [30–32].

In addition to its extreme salt adaptability, *S. alterniflora* is also described as a very good model to study recent allopolyploid speciation [33]. While the present study was in progress, a paper was published on the development of a reference transcriptome of *S. alterniflora* [34]. Some ecologically relevant genes known to be involved in salt and metal stress response were identified from this study, but no effort was made specifically to study its transcriptome that is induced or regulated under salt stress. In the present study, we performed a genome-wide transcriptome analysis of *S. alterniflora* with an objective to identify candidate genes on a global scale that possibly play roles in its ability to adapt to extremely saline habitat.

## Methods

### Plant material and RNA isolation

Cuttings of *S. alterniflora* with a single runner were planted in 4-in. plastic pots (one per pot) filled with sand and were placed in a deep plastic container filled with 8 L Hoagland’s nutrient solution. The plants were grown inside a greenhouse maintained at 29/22 °C day/night temperature with 14 h light (200 μM m^-2^ s^-1^). The solution was replenished every week. One-month-old plants with 3–4 leaves were subjected to salinity stress (500 mM NaCl). Leaf and root tissues were harvested in liquid nitrogen from plants before salt stress (control) and after 6, 12, 24 and 72 h of salt stress, and stored at -80 °C for RNA extraction. Three plants (biological replicates) were sampled for each time point.

### RNA extraction, library preparation, sequencing and assembly

Total RNA was purified from the control and salt-stressed leaf and root tissues using RNeasy plant minikit (Qiagen, Valencia, CA). RNA from different time points (2 μg each) were pooled for leaf (stressed leaf—LS) and root (stressed root—RS) separately. Ten μg of RNA from SL, SR, and control leaf (LC) and root (RC) tissues were subjected to RiboMinus kit (Invitrogen, Carlsbad, CA) to deplete the ribosomal RNA. Complementary DNA was synthesized from ribominusRNA and then normalized using a Trimer-2 normalization kit (Evrogen, Russia) to minimize representation of commonly abundant transcripts. A total of 500 ng of normalized cDNA were sequenced in two half plates on the Roche’s 454 GS-FLX platform (MoGene, St. Louis, MO).

Sequences were cleaned for 454 sequence primers midi tags used for multiplexing using an in-house Perl script. Low quality sequences (score < 20) and reads matching to noncoding RNAs, such as tRNA, and miRNA and snRNA, predicted using tRNAscan-SE tool with eukaryote parameters and RFAM v10.1 covariance models in INFERNAL format (http://rfam.xfam.org/), were also removed prior to further analysis. The remaining reads were assembled using CAP3 [35], separately for four libraries, LC, LS, RC and RS with default parameters where 90 % minimum match on at least 100 bp was executed that allowed a perfect match to satisfy the minimum alignment score for assembly. The stringency of assembly was kept low as suggested by [34] to assemble possible homologous reads to construct consensus contigs to constitute de novo transcriptome with expectedly up to six allelic transcripts per locus for the hexaploid grass. Assembly was validated by comparing the sequence identity of the de novo contigs against homologous ESTs reported earlier [29]. Singletons less than 50 bp were not included in further analysis. Also, realignment of the sequence reads against the assembled unigenes was performed as a further validation using Bowtie version 1.1.0 [36], which supports realignment of reads up to 1024 bp, with three mismatches and no gaps.

### Functional annotation

*Spartina alterniflora* unigenes (contigs and singletons) were functionally annotated by sequence similarity search against NCBI protein and nucleotide (http://www.ncbi.nlm.nih.gov/), SwissProt and TrEMBL (http://www.uniprot.org/help/uniprotkb) non-redundant (nr) plant sequence database using BLASTx and BLASTn algorithm at 1e-06 threshold. Top hits based on significant match of the alignments were parsed using in-house Perl script into MySQL database. The linear regression analysis for finding the relation of length and annotation was performed using SAS v.9.3 (SAS, 2011). Further, based on the significant hits obtained with NCBI nr database, the pathways in which genes are involved were retrieved from the Kyoto Encyclopedia of Genes and Genomes (KEGG) pathway database. The gene ontology (GO) terms and IDs of the functionally annotated *S. alterniflora* unigenes were assigned on the basis of BLAST against plant GOslim set and compared with *Oryza sativa* GOslim terms using GOslim viewer (http://agbase.msstate.edu/cgi-bin/tools/goslimviewer_select.pl). The GOslim enrichment analysis was performed using BinGO tool [37] and visualized using Cytoscape. The hypergeometric test with false discovery rate (FDR) correction methods [38] was used for GO enrichment analysis to assess overrepresentation of GOslim categories.

Protein families represented in the *S. alterniflora* transcriptome were predicted by HMMsearch program of HMMER v3.0 against the hidden Markov models (HMMs) Pfam-A v26.0 database (http://pfam.xfam.org/) at e-value threshold 1.0. The database contains a set of high quality, manually curated and annotated models, and is considered as a good source for finding protein families [39, 40]. The output obtained from HMM search was parsed by an in-house Perl script and further analysis was performed using MySQL.

### Syntenic block analysis

To find the syntenic block of the *S. alterniflora* transcriptome relative to rice chromosomes, we performed the BLASTn search against all 55,986 gene loci of the rice transcriptome database (rice genome annotation project release 7) at 1e-05 threshold. The sequences showing identity greater than 60 % were considered as syntenic block. Further, chromosomal location of the syntenic blocks was assigned based on the physical location of these sequences on the respective rice chromosomes. A karyotype file was generated using an in-house Perl script for Circos plot of rice chromosomes and MySQL database was used for generating the connecting lines (link) to represent similar blocks between *S. alterniflora* and rice, and color-highlighting syntenic regions (input) on rice chromosomes. A comparative GC content analysis of *S. alterniflora* transcriptome relative to rice transcriptome was performed using in-house Perl script.

### Identification of gene families

For defining the gene families of *S. alterniflora*, its transcriptome was compared with the transcriptomes of three monocots – *Oryza sativa* (*Os*), *Zea mays* (*Zm*) and *Sorghum bicolor* (*Sb*) individually and together in groups. The coding sequences of the three monocot species were downloaded from the https://phytozome.jgi.doe.gov/pz/portal.html (v8) for BLASTn analysis at 1e-20 with at least 60 % similarity in the aligned region. The results were clustered using Markov Cluster algorithm [41] with default inflation (I) value of 6 to define gene families. The 4-way Venn diagram representing gene families from all four species was created using R package.

### Identification of lineage- and species-specific genes

Lineage- and *S. alterniflora*-specific genes were identified by sequential comparative similarity searches within the nucleotide and protein sequences available in plant kingdom to exclude the sequences conserved outside the family poaceae and within poaceae. BLASTx, and BLASTn algorithms were used for stepwise search at threshold e-value 1e-01. The potential reason for keeping low e-value cut-off is due to comparatively large number of smaller sequences in *S. alterniflora* transcriptome. First, all sequences of *S. alterniflora* were searched against all non-poaceae species proteome dataset available at phytozome v10 (https://phytozome.jgi.doe.gov/pz/portal.html) with BLASTx algorithm. The sequences that showed significant hit to given non-poaceae peptide sequences were filtered out for further analysis. The remaining sequences were searched against PlantGDB (http://www.plantgdb.org), UniProtKB (http://www.uniprot.org) and NCBI nr/nt and unigenes (http://www.ncbi.nlm.nih.gov) for non-poaceae species. The transcripts with significant hit to these databases were filtered out. The remaining genes, which are putative members of poaceae and *Spartina* specific groups, were further analyzed with poaceae family genes from same database as mentioned above. The genes showing significant hits to the poaceae database were filtered out and grouped into poaceae-specific genes. Thus, the remaining transcripts that did not show any hit to any database mentioned above were termed as *Spartina*-specific genes. The filtering of the sequences at different stages was carried out using custom Perl script and MySQL. The GC content analysis was performed by custom Perl script.

### Identification of transcription factors (TFs), protein kinases, and transporters

For the identification of TFs in *S. alterniflora*, its unigenes were BLAST searched with 1e-05 threshold and aligned region identity of 80 % against all 29,473 protein sequences representing TFs and transcription regulators within 84 families of major plant species available at plant transcription factors database v3.0 (http://plntfdb.bio.uni-potsdam.de/v3.0/; [42]. The fold change expression of each family member under salt stress relative to control was determined from the normalized transcript abundance based on their reads. The differentially up- and downregulated TFs were represented in a heat map as described earlier [43]. Similarly, comparative analysis of *S. alterniflora* unigenes similar to kinases was performed with kinome from rice phylogenomic database (http://ricephylogenomics.ucdavis.edu/kinase/genInfo.shtml; [44]. The protein phosphatases in *S. alterniflora* transcriptome were analyzed through domain search by InterProScan (v5) using PFAM and SMART database. For comparative analysis with rice, the phosphatase domain of rice transcriptome was retrieved from the rice genome project database v7.

### Differential gene expression analysis

Gene expression in *S. alterniflora* leaf and root under salt stress was deduced from the abundance of transcripts (number of reads per contig) in comparison to unstressed control condition. The sequence reads were mapped to the rice genome sequence using BWA-SW and reads belonging to each gene model were counted. The read counts under salt and control were normalized and used to identify differentially expressed genes (DEGs) using DEGseq v1.2.2 [45]. Genes with log2 fold change ≥ 2 and ≤ - 2 (*P* < 0.05) were categorized as significantly differentially expressed genes. The DEGs were visualized using MA plot between M (log2 fold change) and A (average of log2 fold change between control and salt stress). Validation of the digital expression analysis was performed using (semi)quantitative RT-PCR analysis as detailed earlier [30].

### Identification of simple sequence repeats and genic primer analysis

Unigenes of *S. alterniflora* were searched for simple sequence repeat (SSR) motifs using MIcroSAtellite Perl script (MISA, http://pgrc.ipk-gatersleben.de/misa). Unigenes with SSRs of at least six for di, and five for tri, tetra, penta and hexa-nucleotides were used to design ESSR primers as described earlier [27]. The positions of the SSRs on open reading frames of the genes were determined by using gene finding software MolQuest (FGENESH+; http://linux1.softberry.com/berry.phtml). Predictive function of the sequences containing SSR motifs were retrieved from the MySQL database containing information on functional annotation of unigenes. SSRs with mononucleotide tandem repeats were not considered for downstream analysis, and SSRs with at least six contiguous repeats of di-, tri-, tetra-, penta-nucleotide and five repeats for hexa-nucleotides were used for design of SSR primers. Six SSR primers were tested in a set of 13 *S. alterniflora* accessions (CP1 through CP13) using the PCR conditions and profile as described by [27].

## Results and discussion

### Sequence assembly

**Fig. 1:**
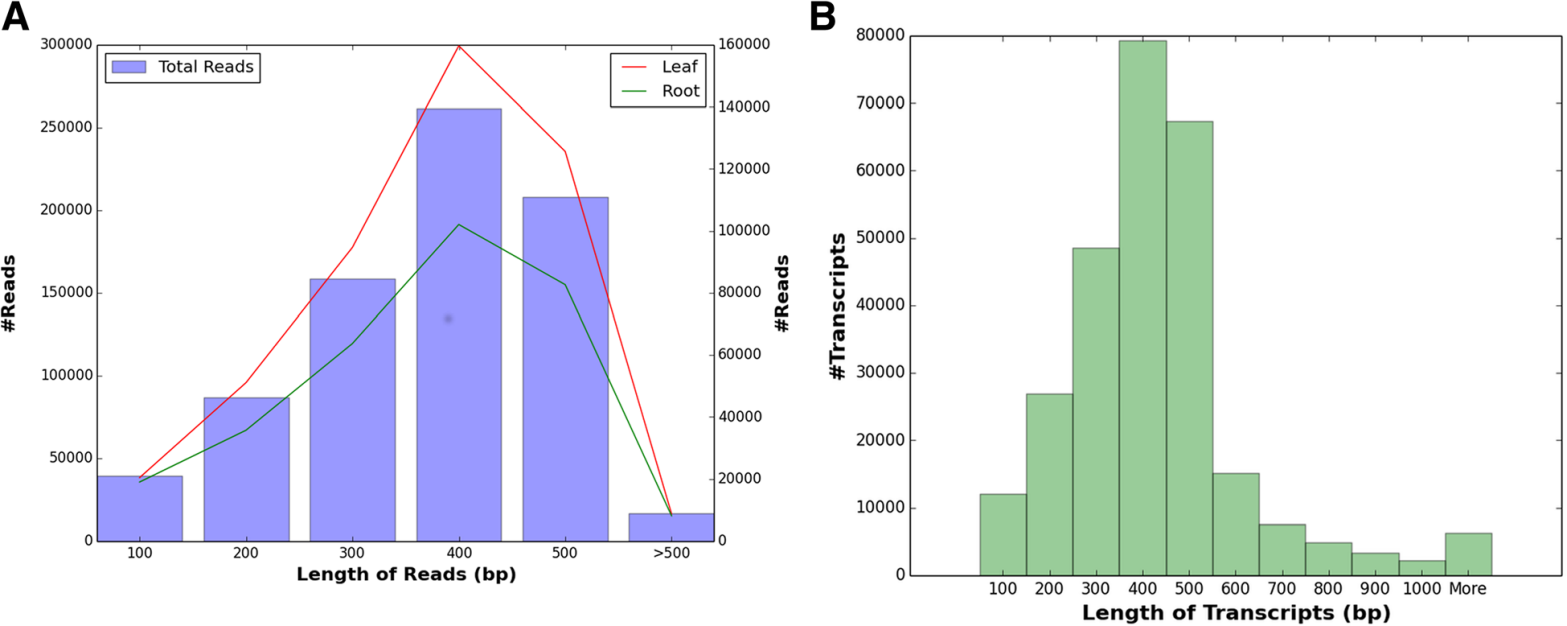
**a** Total number of reads sequenced and their distribution in leaf and root tissue of *Spartina alterniflora*; **b** Histogram showing frequency distribution of length of *Spartina alterniflora* unigenes

A total of 770,690 raw sequencing reads with quality score of 20 (Q20) were generated from the leaf and root tissues of *S. alterniflora* with and without salt stress. The raw sequence reads are available in the NCBI SRA database under the bioproject PRJNA302213 (http://www.ncbi.nlm.nih.gov/bioproject/PRJNA302231). The length of the reads ranged from 16 to 616 bp with an average length of ~324 bp (Table 1), spanning 249.6 Mbp of the transcriptome. The leaf and root tissues constituted 59.7 and 40.3 % of the total reads, respectively (Fig. 1a). The high quality reads were assembled into 73,131 contigs and 200,329 singlets (more than 50 bp), which totaled to 273,460 unigenes (Table 1). The leaf tissue accounted to 36.6 % of contigs and 63.4 % of singlets, whereas the root tissue accounted to 19.2 % of contigs and 83.9 % of singlets combined under stress and control conditions (Table 2). Short read sequence data from 454 pyrosequencing have been successfully assembled and the resulting contigs have been explored for transcriptome analysis of non-model plants like *S. alterniflora* [34]. Larger number of singletons as compared to contigs in the present study could be attributed to assembly algorithm used, low-expressing transcripts, repeat regions in the reads, artifacts in cDNA normalization, sequencing error, and/or possible contamination with other organisms [46]. But, the high match of singletons to the NCBI protein database suggested that singletons were of high quality and not the result of sequencing errors, and were likely derived from the low-expressing transcripts. In addition, realignment of 85.8 % unigenes to at least one read of the transcriptome of *S. alterniflora* validated the quality the transcriptome. The length of the contigs ranged from 51 to 6460 bp with an average of 577 bp, whereas the length of singlets ranged from 51 to 616 bp with an average of 316 bp; the average length of unigenes was 386 bp (Fig. 1b). The N50 and N90 values were 317 bp and 255 bp, respectively. Most (79,295) of the unigenes were between 401 to 500 bp in length. The unigenes covered 105.5 Mbp of *S. alterniflora* transcriptome. Considering that 10 % of its genome (1,763.9 Mbp; [27] represented transcribed region, the transcriptome coverage was 0.6X, but with a sequence coverage of 5.9X. Although the depth of coverage is low in 454 sequencing, it has the capability to capture rare transcripts because of its sequence length coverage (386 bp average unigene size in this study), and has been a powerful sequencing technology in the absence of a reference genome [47]. Interestingly, only 4.3 % of the reads from this study had 100 % match to previously published reads of *S. alterniflora* [34], but 41.1 % reads showed more than 90 % match (Additional file 1).

**Table 1:** Assembly and annotation of *Spartina alterniflora* transcriptome

Parameter	Statistics
Number of raw reads	770,690
Number of reads in leaf tissue	459,819
Number of reads in root tissue	310,871
Total span of reads	249.59 Mbp
Average length of reads	~324 bp
Number of Unigenes (50 bp or greater)	273,460
Number of Contigs (50 bp or greater)	73,131
Large Contigs (500 bp or greater)	35,519
Large Unigenes(500 bp or greater)	39,682
Number of Singletons (50 bp or greater)	200,329
Number of Unigenes (Leaf Control)	73,552
Number of Unigenes(Leaf Stress)	68,346
Number of Unigenes (Root Control)	72,199
Number of Unigenes (Root Stress)	59,363
Total span of Contigs	42.19 Mbp
Total Span of Singletons	63.29 Mbp
Transcriptome Size	105.48 Mbp
N50 length	52.74 Mbp
N50 Value	317 bp
N90 length	94.93 Mbp
N90 Value	255 bp
Longest Unigene	6.46 Kb
Smallest Unigene	51 bp
Mean length of Unigenes	~386 bp
GC content (%)	51.46
Unigenes with significant hit (%)	187,571 (68.59 %)
Unigenes with unknown functions	9,684 (5.16 %)
Unigenes having unique protein annotation	47,960 (25.56 %)

**Table 2:** Sequencing and CAP3 assembly statistics of *Spartina alterniflora* transcriptome

Library	Total no. of reads sequenced	Reads assembled into contigs	Reads as singlets	% redundancy^a^	Total unigenes
Leaf Control	239,851	27,691	45,861	80.87 %	73,552
Leaf Stress	219,968	24,237	44,109	79.94 %	68,346
Root Control	215,127	15,792	56,407	73.77 %	72,199
Root Stress	95,744	5411	53,952	43.64 %	59,363
Total	770,690	73,131	200,329		273,460

^a^Redundancy calculated as the number of reads assembled/total number of reads

### Functional annotation and GOslim analysis

**Fig. 2:**
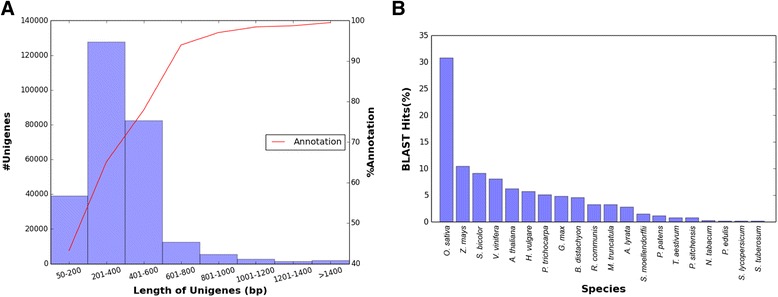
**a** Total number of *Spartina alterniflora* unigenes and their functional annotation obtained using similarity search against NCBI and UniProt database; **b** Distribution of the species with genes that were highly homologous to *Spartina alterniflora* unigenes based on the significant hits obtained from BLAST analysis

BLASTx and BLASTn searches of the unigenes against UnitprotKB and NCBI nucleotide database (nr) showed match to total 187,571 (68.6 %) unigenes of which 61,851 (33.0 %) and 125,720 (67.0 %) were contigs and singlets, respectively. Of the total 187,571 hits, 47,960 (25.6 %) had unique hits represented in the respective database. Most (94.8 %) of the unigenes had annotation to proteins of known function; however, 9579 (5.1 %) and 105 (0.1 %) had hit to hypothetical and unknown proteins, respectively. The low percentage (68.6 %) of annotation was attributed to the large number of short singleton sequences present in the dataset. Of the total unigenes, 84.9 % contigs were annotated while only 62.8 % singlets had annotation. The linear regression analysis (Fig. 2a) showed that *S. alterniflora* unigene length was a significant predictor of annotation (*R*^2^ = 0.78, *P* < 0.0036). This situation was also evident in root tissues where 83.9 % of the transcripts were singlets, and overall, 56.4 % had BLAST matches in comparison to 79.9 % transcripts (63.4 % singlets) in leaf tissue (Table 2). High percentage of unigenes (92.2 %) with length ≥ 500 bp had significant BLAST hits as compared to 64.6 % unigenes having length < 500 bp. These results are in agreement with previous reports that longer sequences were more likely to annotate using BLAST searches with protein database than shorter sequences [46, 48, 49].

Over 30 % of the *S. alterniflora* unigenes showed more than 90 % similarity with genes of *Oryza sativa* followed by *Zea mays*, *Sorghum bicolor*, *Vitis vinifera*, and *Arabidopsis thaliana* (Fig. 2b). *S. alterniflora* shares significant similarity with rice at both DNA and protein levels [29, 50]. Therefore, rice transcriptome and genome were used as references for comparative studies of the *S. alterniflora* transcriptome. Only 1.5 % of the unigenes showed homology with bacterial proteins, which could be due to contamination of the tissue samples, especially root. The transcripts that did not have any annotation could represent species-specific genes, untranslated regions (UTR), non-coding RNA and/or genes with novel uncharacterized function. The average GC content of *S. alterniflora* transcripts (51.5 %) was comparable to that of rice (51.3 %; Additional file 2: Figure S1).

**Fig. 3:**
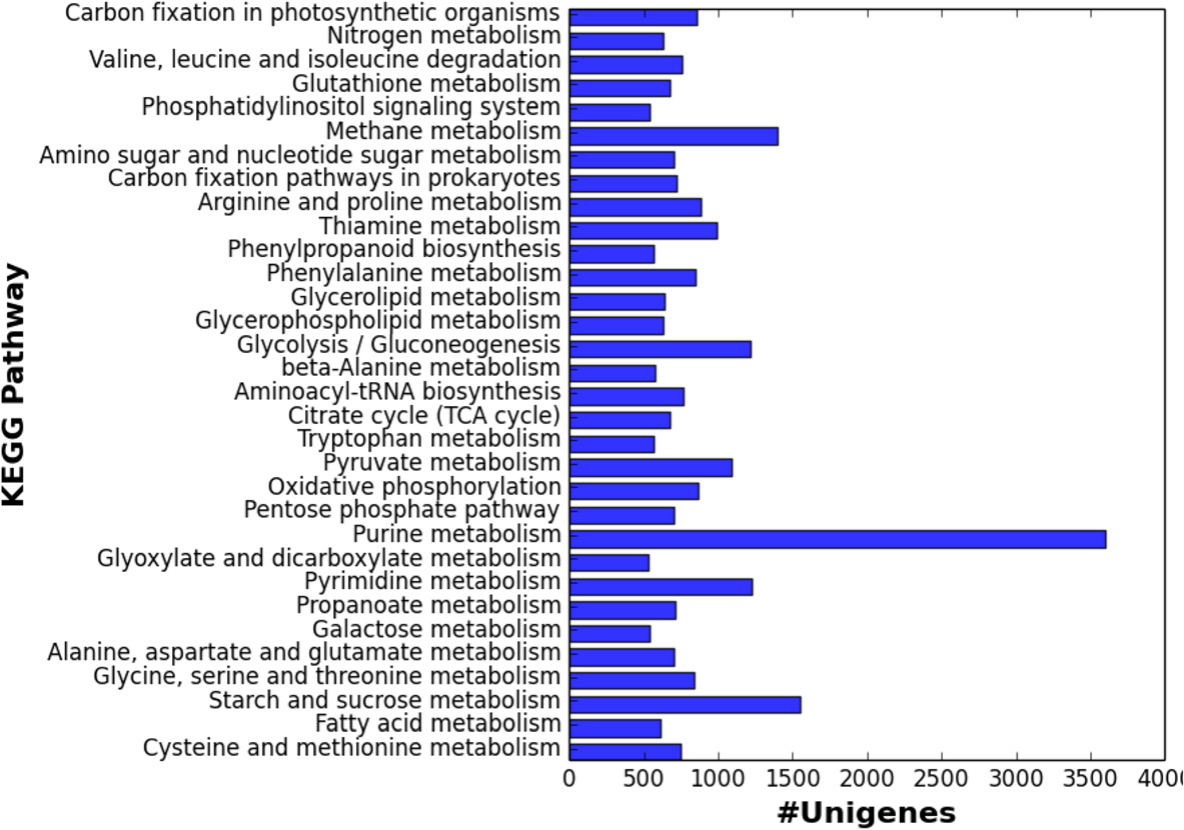
Highly expressed biological pathways represented in *Spartina alterniflora* transcriptome retrieved from the KEGG database

Based on the similarity to HMM profile of protein domains of Pfam-A database, 21,121 (7.7 %) *S. alterniflora* unigenes were assigned to 6729 unique protein families (Additional file 3: Table S1). The KEGG pathway annotation of the transcripts revealed the diversity of pathway, molecular interaction in the cells and biological functions represented in the *S. alterniflora* transcriptome. Highly expressed KEGG pathways were represented by the transcripts involved in metabolic processes, such as amino acid metabolism, carbohydrate metabolism, fatty acid metabolism, and nitrogenous compound metabolism of which purine, methane and carbon metabolic processes were dominant (Fig. 3; Table 3). Phenylpropanoid biosynthesis and amino acid degradation represented major secondary metabolism pathways (Fig. 3).

**Table 3:** The enzymes and their IDs for the *Spartina alterniflora* transcripts involved in highly expressed pathways

Enzyme	ID
L-amino-acid oxidase	ec:1.4.3.2
3-hydroxybutyryl-CoA epimerase	ec:5.1.2.3
maltose alpha-D-glucosyltransferase	ec:5.4.99.16
homoserine kinase	ec:2.7.1.39
L-amino-acid oxidase	ec:1.4.3.2
6-phosphofructokinase	ec:2.7.1.11
methylmalonyl-CoA epimerase	ec:5.1.99.1
uridine kinase	ec:2.7.1.48
methylmalonyl-CoA epimerase	ec:5.1.99.1
5-(carboxyamino)imidazole ribonucleotide mutase	ec:5.4.99.18
2-dehydro-3-deoxygluconokinase	ec:2.7.1.45
succinate dehydrogenase (ubiquinone)	ec:1.3.5.1
pyruvate kinase	ec:2.7.1.40
L-amino-acid oxidase	ec:1.4.3.2
succinate dehydrogenase (ubiquinone)	ec:1.3.5.1
L-seryl-tRNASec selenium transferase	ec:2.9.1.1
malonyl-CoA decarboxylase	ec:4.1.1.9
pyruvate kinase	ec:2.7.1.40
choline kinase	ec:2.7.1.32
glycerate kinase	ec:2.7.1.31
L-amino-acid oxidase	ec:1.4.3.2
4-coumarate---CoA ligase	ec:6.2.1.12
hydroxymethylpyrimidine kinase	ec:2.7.1.49
oxaloacetate decarboxylase	ec:4.1.1.3
methylmalonyl-CoA epimerase	ec:5.1.99.1
L-arabinokinase	ec:2.7.1.46
glycerone kinase	ec:2.7.1.29
CDP-diacylglycerol---inositol 3-phosphatidyltransferase	ec:2.7.8.11
gamma-glutamylcyclotransferase	ec:2.3.2.4
methylmalonyl-CoA epimerase	ec:5.1.99.1
D-amino-acid dehydrogenase	ec:1.4.99.1
pyruvate kinase	ec:2.7.1.40

**Fig. 4:**
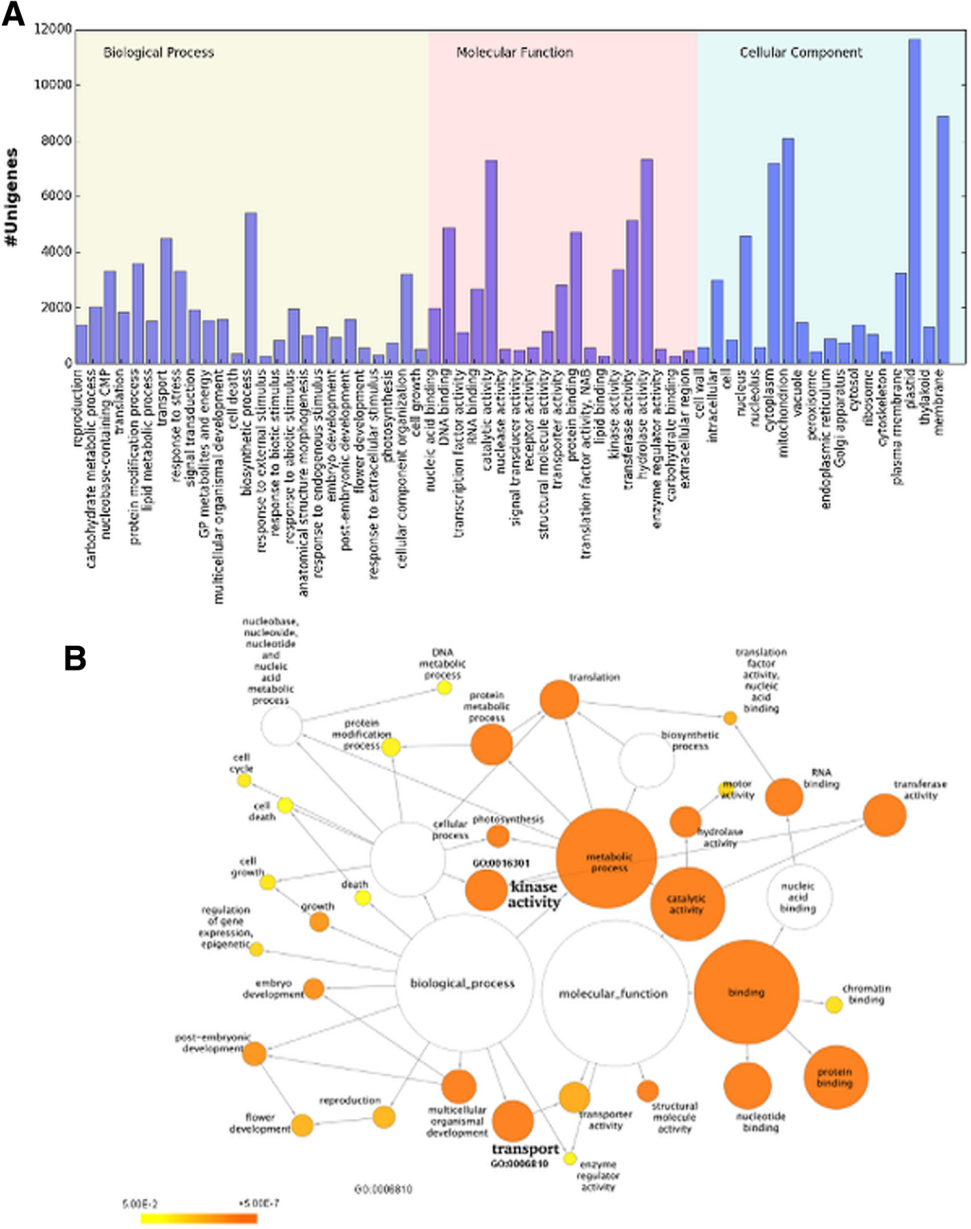
**a** GOslim functional analysis of *Spartina alterniflora* genes showing their distribution in biological process, cellular component and molecular function. **b** Enrichment of specific GOslim terms induced under salt stress in the transcripts from leaf and root tissue

The gene ontology analysis (GOslim terms) assigned 57.0, 50.0, and 43 % of *S. alterniflora* transcripts to biological process, molecular function, and cellular component, respectively. *S. alterniflora* unigenes covered wide range of functional GO categories (Fig. 4a). GOslim terms for biosynthetic and transport processes were highly represented in the biological processes. Similarly, GOslim terms for catalytic activity and hydrolase were higher in the molecular function category. Plastid was the highly presented GOslim term under cellular component category. Analysis of the GOslim terms of *S. alterniflora* transcripts showed that genes involved in transport and kinase activity were highly enriched under salt stress in both leaf and root transcriptome (Fig. 4b).

### Synteny with rice genome

**Fig. 5:**
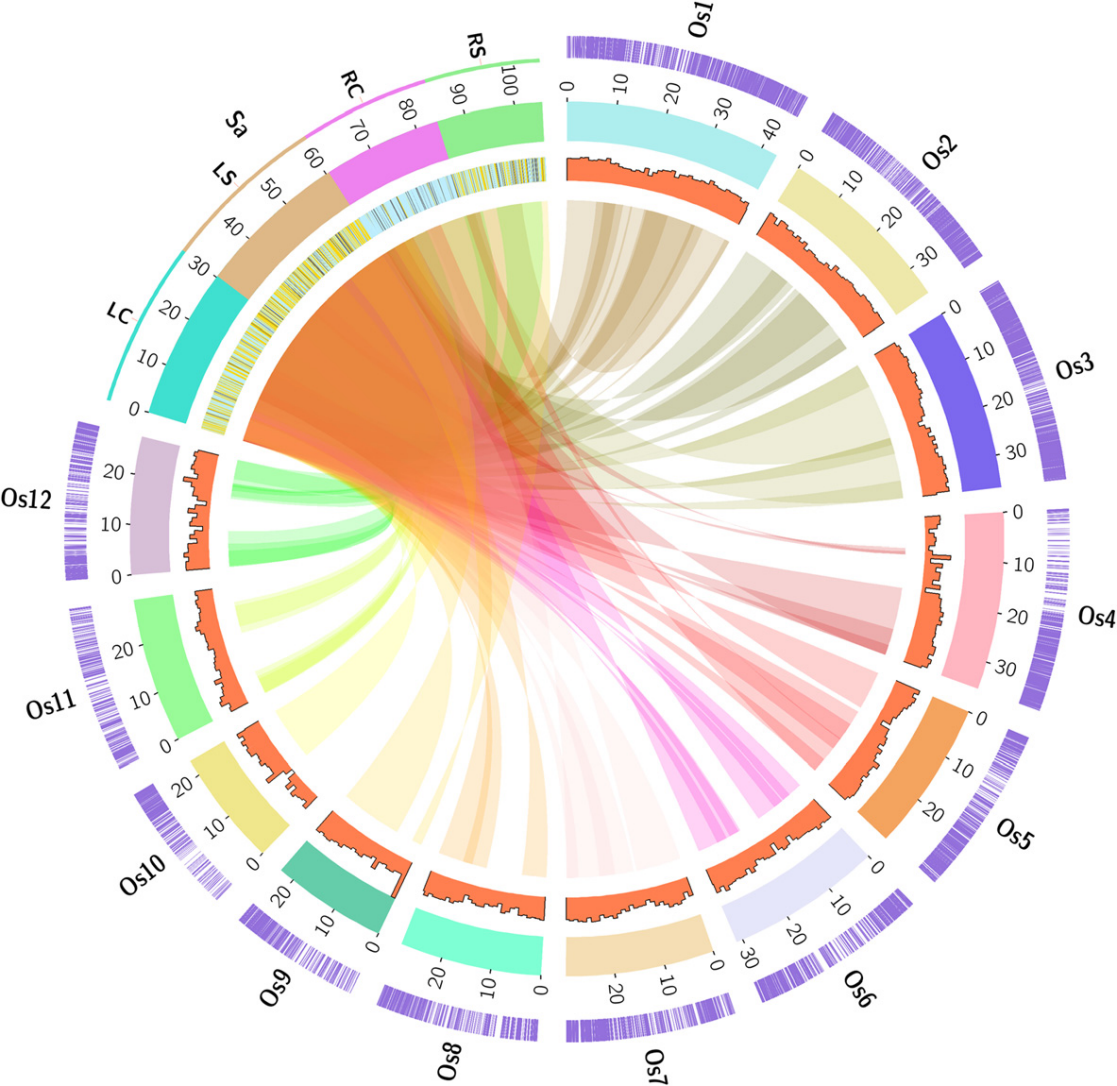
Similarity shared between *Spartina alterniflora* transcriptome and rice genome v7.1. Sa, *Spartina alterniflora*; Os, individual rice chromosomes (different color boxes). Numbers outside the boxes along Os represent the size in megabase (Mbp). Syntenic blocks between Sa and Os are connected by ribbons and highlighted along the Os in purple color. Heatmap representing differential regulation of genes (yellow- upregulated and blue- downregulated) is shown on Sa transcriptome. The gene density of Sa transcripts on rice is shown by histogram

*Spartina alterniflora* showed substantial similarity with rice at both DNA and protein levels (Fig. 5). Homology search of *S. alterniflora* transcriptome was done against rice transcriptome database to find syntenic regions, which showed that ~50 % of the *S. alterniflora* transcripts matched with rice transcriptome at 1e-05 cut off value (Table 4). Distribution of the *S. alterniflora* transcripts along 12 rice chromosomes showed that large numbers of *S. alterniflora* transcripts were represented in chromosome 1 of rice. However, the number of genes of rice identical to *S. alterniflora* transcripts in rice chromosome 1 was less (44.4 %) as compared to chromosome 3 (48.8 %; Table 4). The other chromosomes of rice also had an extensive similarity with *S. alterniflora* transcriptome. This was in congruence with previous results where the comparisons of 10 coding genes between *Spartina* and rice revealed low nucleotide divergence between the two species [50]. Previously we have reported that *Spartina alterniflora*, shares significant similarity with rice at both nucleotide and protein level [28, 29]. This is further evident from the success of using rice oligomicroarrays for gene expression analysis in *Spartina* [50]. Therefore, two subfamily species, rice and *Spartina*, in spite of long period of divergence, apparently have maintained a high level of synteny.

**Table 4:** Mapping of assembed *Spartina alterniflora* transcripts onto rice genome v7.1 (downloaded from rice genome project)

Rice chromosome	Total unique hits of unigenes	Total unigenes spanning rice chromosome	Total no. of genes on rice chromosome	% rice genes covered in rice chromosome	% unigenes covered
Os1	19,652	2898	6529	44.38	7.18
Os2	15,547	2418	5377	44.96	5.68
Os3	17,996	2717	5568	48.79	6.58
Os4	11,413	1968	5317	37.01	4.17
Os5	10,648	1791	4572	39.17	3.89
Os6	10,488	1768	4709	37.54	3.83
Os7	9873	1646	4450	36.98	3.61
Os8	8855	1527	4188	36.46	3.23
Os9	9380	1256	3407	36.86	3.43
Os10	7457	1187	3510	33.81	2.72
Os11	6101	1281	4160	30.79	2.23
Os12	8066	1435	4014	35.74	2.94
Total	135,476	21,892	55,801	39.23	49.54

### Gene families represented in *Spartina alterniflora* transcriptome

Sequence comparison of the *S. alterniflora* transcriptome with transcriptomes of three monocots, such as *O. sativa*, *Z. mays* and *S. bicolor* helped identify different gene families. Similarity search among these Poaceae transcriptomes resulted in a total of 33,142 clusters representing different gene families. Among these, 13,262 clusters representing 158,613 genes were shared by *S. alterniflora* transcriptome with all three transcriptomes. Pair-wise comparison of *S. alterniflora* transcriptome with the three transcriptomes revealed that *S. alterniflora* and *O. sativa* shared maximum number of clusters (2330) containing 9817 genes followed by 1360 clusters containing 4789 genes between *S. alterniflora* and *Z. mays*, and 1046 clusters containing 3668 genes between *S. alterniflora* and *S. bicolor* (Additional file 2: Figure S2). Further, 1366 clusters with 1422 genes were specific to *S. alterniflora* only, which was more than the clusters specific to rice (1307). Similarity search of genes in *S. alterniflora*-specific clusters against UniProtKB database showed that most of the genes were involved in binding, transport and kinases activities (Additional file 2: Figure S3).

### Lineage and species-specific genes in *Spartina alterniflora* transcriptome

The lineage-specific genes share similarity with genes within one taxonomic group and have no similarity with genes outside of a particular taxon [51]. The strategy of identification and characterization of lineage- and species-specific genes is dependent upon the availability of the genomic sequences for that particular species.

**Fig. 6:**
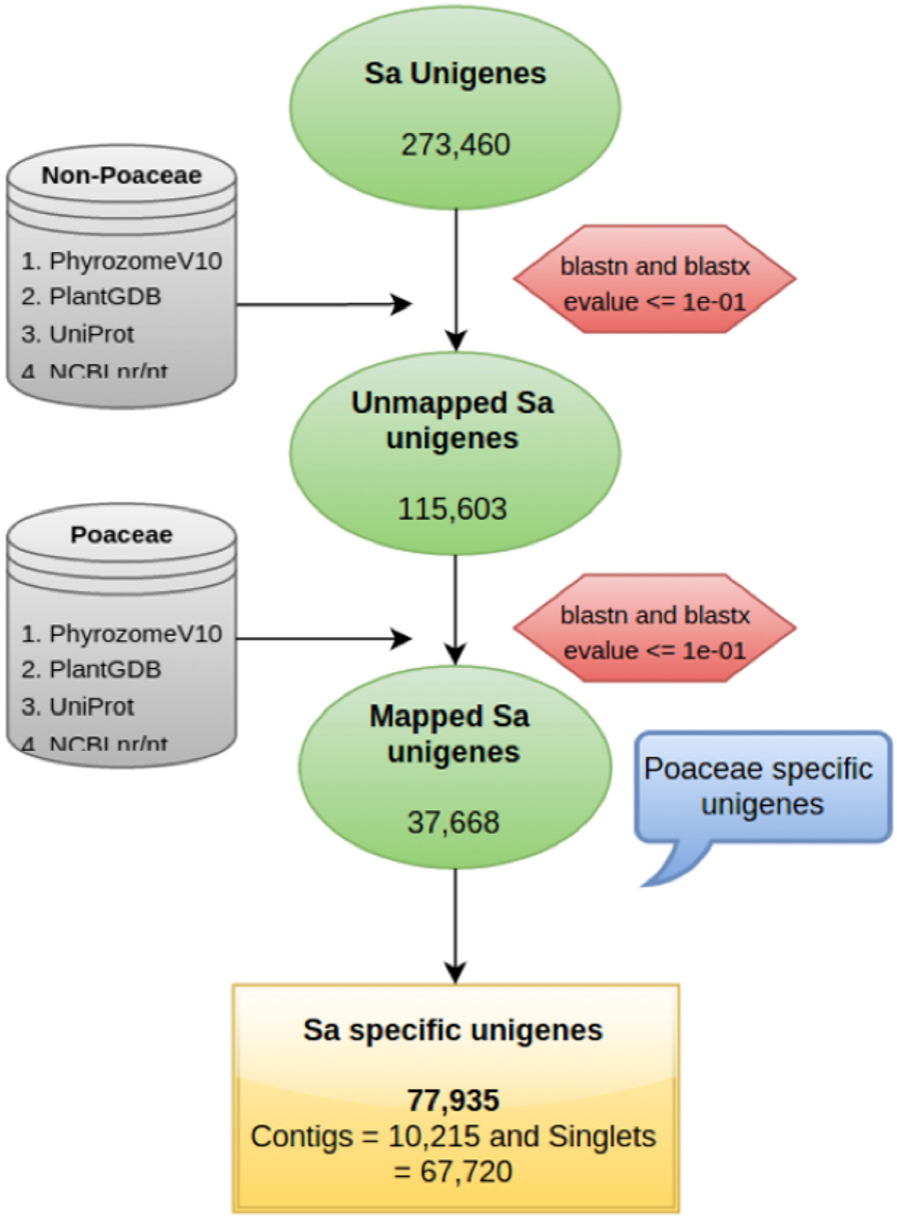
A schematic flowchart showing the strategy followed to determine the lineage specific genes in *Spartina alterniflora* transcriptome

Out of the total 273,460 unigenes of *S. alterniflora*, 157,857 unigenes showed similarity to non-poaceae gene and protein sequences obtained from phytozome, NCBI nr/nt and unigenes databases and UniProtKB. Further analysis of the remaining 115,603 unigenes showed that 37,668 unigenes were poaceae-specific; the remaining 77,935 unigenes (10,215 contigs and 67,720 singlets) were defined as *Spartina*-specific genes in this study (Fig. 6). The large number of sequences specific to *S. alterniflora* in comparison to rice (17.4 %; [52] and *Arabidopsis* (4.9 %; [51] could be due to relatively large number of short-length singletons in *S. alterniflora* transcriptome or its higher genome size compared to rice and *Arabidopsis*. In this study, 13.8 % of *S. alterniflora* transcripts were grouped as poaceae-specific, whereas in rice only 12.5 % of genes were reported as poaceae-specific [52]. In rice, 861 genes were identified to be conserved by evolution within poaceae, but did not share similarity outside the poaceae family. This difference could be due to the larger genome size of *S. alterniflora* compared to rice. Lineage-specific genes are known to have functional and evolutionary significance such as in speciation and adaptation [51, 53].

Distribution of the GC contents for poaceae- and *S. alterniflora*-specific and non-Poaceae transcripts showed that the pattern was similar with the peak range of 48-54 %. However, *Spartina*-specific transcripts did not have a broad range of GC (48.2 %) unlike other transcripts that showed a broad range of 60-70 % (Additional file 2: Figure S4). The results suggested that conserved poaceae-specific genes in *S. alterniflora* followed similar pattern as that in rice. The characterization of the *S. alterniflora*-specific genes will lead to discovery of novel genes with species-specific functions for habitat-adaptive phenotype or traits.

### Differential expression of genes under salt stress

**Fig. 7:**
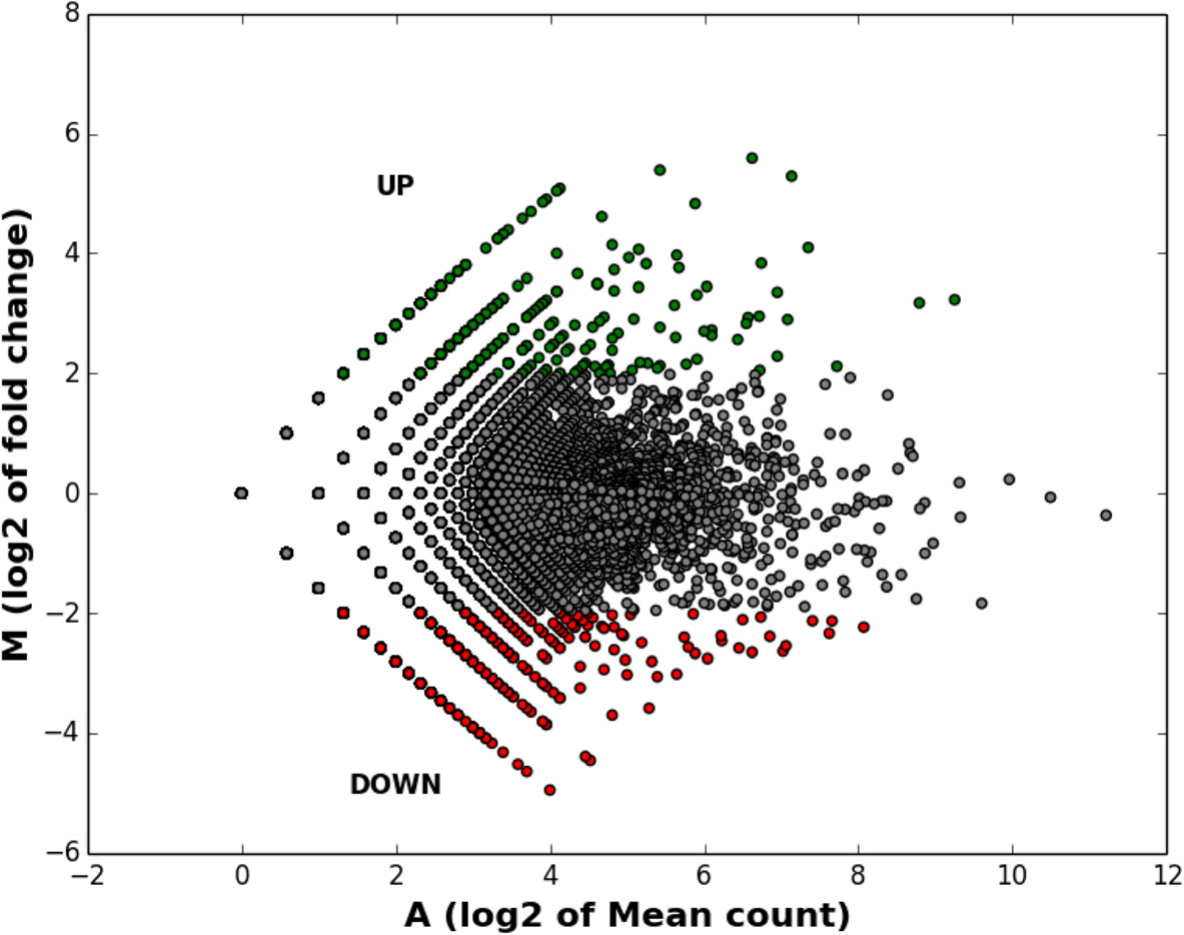
The MA plot showing global differential expression of genes in leaf and root tissue of *Spartina alterniflora*. The *red color* indicates the downregulated genes (log2 fold change ≤ -2) and *green color* indicates upregulated genes (log2 fold change ≥ 2)

The relative expression of transcripts under control and salt conditions in both leaf and root tissues of *S. alterniflora* offered an insight into the potential genes that may possibly be involved in salinity adaptation of the marsh grass. The differential gene expression analysis showed that genes involved in DNA/RNA binding (transcription factors), ion transport and protein kinase activities were upregulated by at least 2-fold (log2 of fold change) under salt compared to control (Fig. 7).

### Transcription factor encoding genes

Stress response in plants is dependent on the transcriptional control of stress responsive genes [54]. Identification and characterization of transcription factors overexpressed under salt stress will be helpful to elucidate the salt stress tolerance mechanisms in *S. alterniflora* and other halophytes towards engineering of the salt sensitive crops for improved salt stress tolerance [55]. Several salt responsive transcription factors were reported in previous studies based on the small-scale EST sequencing of *S. alterniflora* [28, 29]. Based on the search against all 84 families of transcription factors including transcription regulators in plant transcription factor database, 4462 (1.63 %) unigenes belonging to 54 TF families and 22 other transcription regulators (TR) were identified in *S. alterniflora* leaf and root transcriptome.

**Fig. 8:**
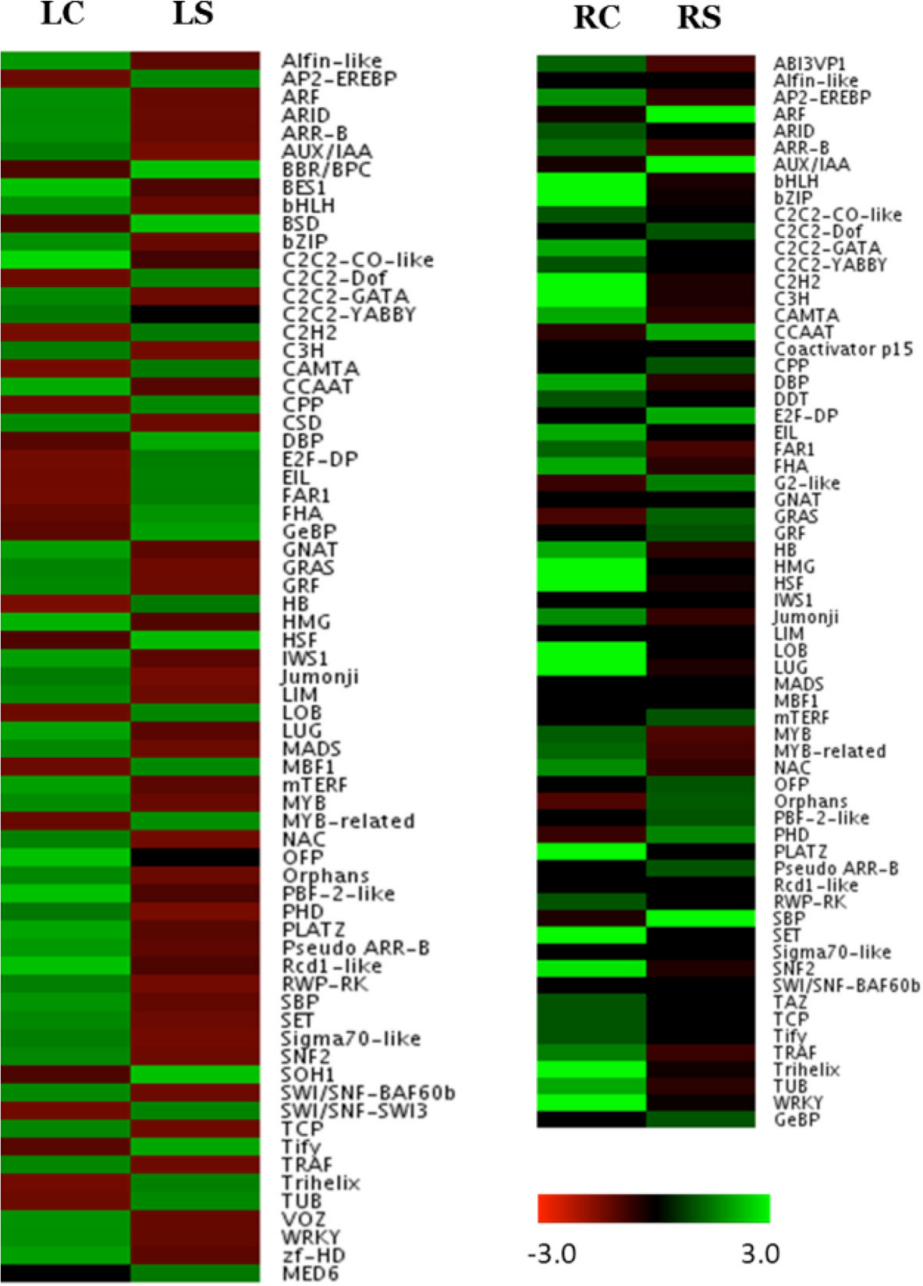
Expression of transcription factors/regulators in leaf and root tissue under control and salt stress condition of *Spartina alterniflora*. The rows indicate specific TFs/TRs and columns specify the experimental conditions. Individual cell represents up-regulation (*green*) or down-regulation (*red*) of the genes. LC, leaf control; LS, leaf stress; RC, root control; RS, root stress

Among the 76 families of TFs and TRs, single C(2)-C(2) zinc finger-DNA-binding with one finger (C2C2-Dof) domain proteins, cysteine-rich polycomb-like protein (CPP), E2F-DP and GL1 enhancer binding protein (GeBP) transcription factors were highly expressed under salt in both leaf and root tissues of *S. alterniflora*. Of the 24 TFs/TRs that were upregulated under salt stress in leaf tissue, HSF, BBR/BPC and BSD TFs were significantly upregulated. In root, among the 17 upregulated TFs/TR under salt stress, ARF, AUX/IAA, CCAAT, E2F-DP and squamosa-promoter binding protein (SBP) were significantly enriched (Fig. 8). HSFs were highly induced in *Arabidopsis* by abiotic stresses, such as cold, salinity and osmotic stress [56]. AP2-EREBP and MYB-related TFs are known for their role in salt stress response [57–60]. The expression of ARF and AUX/IAA was reported to be upregulated under salt stress in sorghum [61]. TFs, such as HSF, BBR/BPC and BSD, which were significantly upregulated in the leaf tissue, were downregulated in root under salt stress, thus implying their tissue-preferred expression. Significantly upregulated salt-induced TFs in leaf and/or root tissues suggest their possible role in salt stress adaptation of *S. alterniflora* and calls for their functional characterization to implicate their possible use in regulon engineering for tolerance to high salinity in plants.

### Salt induced transporters

**Fig. 9:**
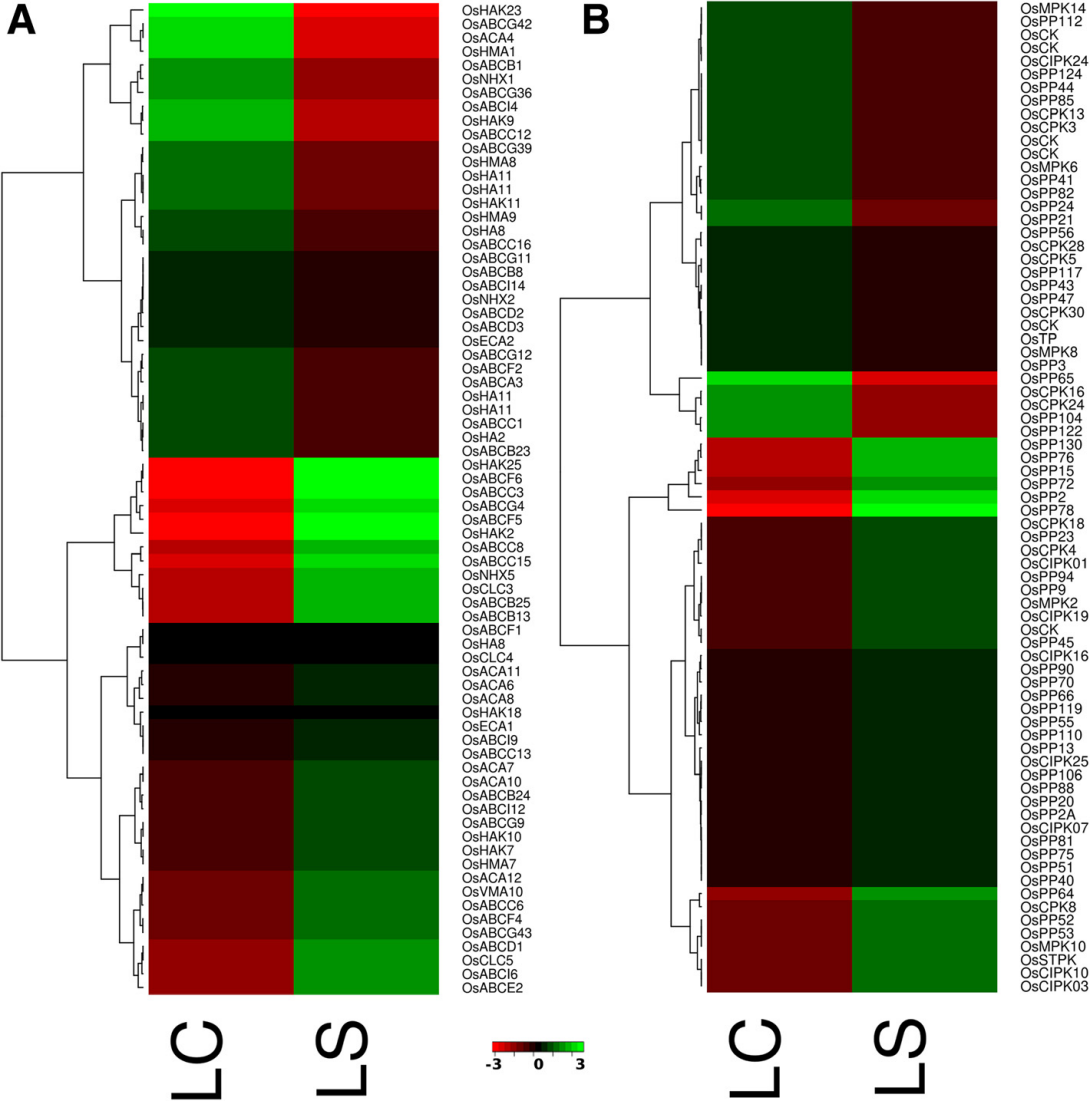
Expression of transporters (**a**) and phosphatases/kinases (**b**) in leaf tissue under control and salt stress condition of *Spartina alterniflora*. The rows indicate specific transporters/phophatases/kinases and columns specify the experimental conditions. Each individual cell represents up-regulation (*green*) or down-regulation (*red*) of the genes. LC, leaf control; LS, leaf stress

To sustain under high salinity, plants maintain ion homeostasis. Salt tolerance in halophytes is dependent upon regulated uptake and accumulation/compartmentalization of Na^+^, K^+^ and Cl^-^ [12, 62]. The regulation and maintenance of high K^+^/Na^+^ level is necessary for the halophytes to cope with salt stress and for normal plant growth [63]. The uptake of salt and its transport through cell is regulated by various transmembrane proteins. Plants maintains the Na^+^ and K^+^ level by employing plasma membrane and vacuolar Na^+^/H^+^-antiporters and high- and low-affinity K^+^ channels [63]. In *S. alterniflora* in the present study, potassium transporter and Na^+^/H^+^ antiporter, hydrogen ion transporters were highly upregulated under salinity. Several plants overexpressing Na^+^/H^+^ transporters have shown improved salt tolerance (reviewed in [55]). Na^+^/H^+^ antiporter helps in maintaining low concentration of Na^+^ by active exclusion of Na^+^ ions into vacuole and apoplast [64]. This secondary transport is energized by the membrane potential and proton gradient generated by the plasma membrane H^+^-ATPase and vacuolar H^+^-ATPase [12]. In halophytes and other salt tolerant plants, high salinity causes an increase in the activity of V-type and P-type ATPases (reviewed in [55]). The activity of P-type ATPases was increased under salt stress in an extreme halophyte *Salicornia brachiata* [65]. Increase in V-type H^+^-ATPase activity and H^+^ transport was also observed under salt stress in sunflower roots [66]. Similar results were obtained in the present study where increased abundance of Na^+^/H^+^ antiporter, P-type and V-type ATPase transcripts were observed in the leaf tissue of *S. alterniflora* under salt stress (Fig. 9a).

Potassium transporters and ABC transporters were also overexpressed under salt stress in the leaf tissue of *S. alterniflora*; chloride transporters were also up-regulated in leaf under salt stress (Fig. 9a). ABC transporters were reported to be up-regulated under salt stress in rice [63]. Cl^-^ is the balancing anion for Na^+^ and it has an equivalent role to that of Na^+^ in salt tolerance of halophytes [62]. These findings suggest that halophytes, such as *S. alterniflora*, maintain their salt tolerance ability by regulating uptake and accumulation of Na^+^, K^+^ and Cl^-^ through upregulation of selective stress-related transporters.

### Phosphatases/kinases

Differential gene expression analysis showed that CDPK/SnRK family of protein kinases, Casein kinase (CK) family, cyclin-dependent kinase family, serine-threonine protein kinase family, Mitogen activated protein kinases (MAPK) family, CBL protein kinase, and protein phosphatase family genes were up-regulated under salt stress in *S. alterniflora* (Fig. 9b) by log2 fold change of ≥2. Ca^2+^ signaling under the regulation of Ca^2+^-dependent kinases plays an important role in controlling cellular processes in response to abiotic stresses such as drought, cold and high salinity [67, 68]. Similarly, expression of mitogen activated protein kinases (MAPK) has been shown to improve abiotic stress tolerance in plants [69]. Protein phosphatases, such as PP2C, were up-regulated by log2 fold change ≥2 in *S. alterniflora* under salt stress (Fig. 9b). The conserved domain analysis using PFAM and SMART database revealed that only 113 out of 1655 *S. alterniflora* transcripts contained the phosphatase domain, and PP2C was the major class of protein phosphatase gene in *S. alterniflora*; similar result was reported in rice [70].

**Fig. 10:**
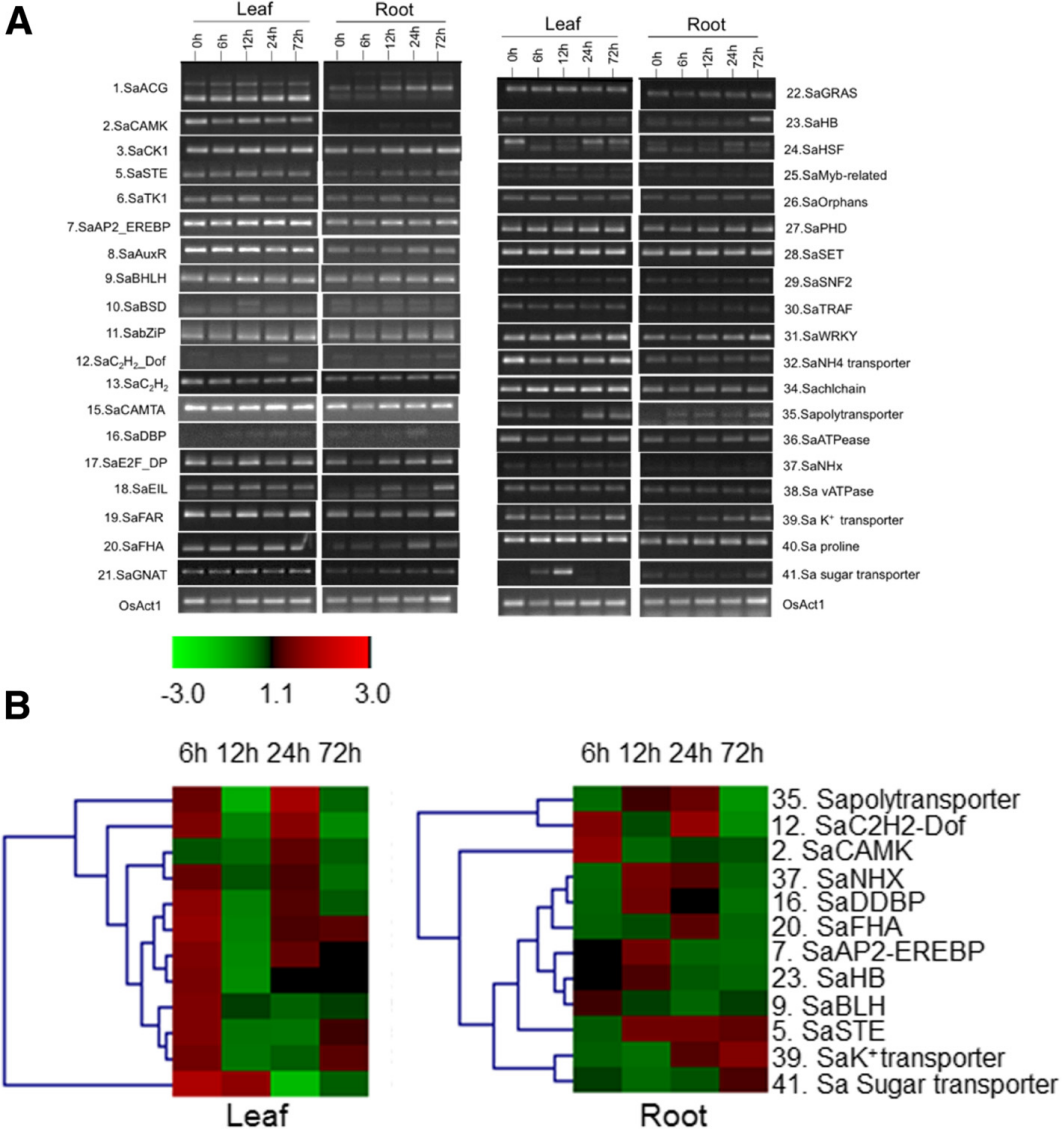
**a** Semiquantitative reverse transcription PCR (SqRT-PCR) and **b** heatmap of expression of a selected set of genes under control and different time points under salt stress in leaf and root tissue of *Spartina alterniflora*

Heat shock proteins (HSPs) and heat shock factors (HSFs) were among other genes that were upregulated (log2 fold change ≥ 2) in *S. alterniflora* under salt. HSPs, HSFs and protein phosphatases are known to be differentially regulated under salt stress [56, 70, 71]. Semiquantitative RT-PCR analysis and heatmap of a selected set of genes representing different groups validated their time and tissue-dependent differential expression under salt stress (Fig. 10a and b).

### Microsatellite analysis

**Fig. 11:**
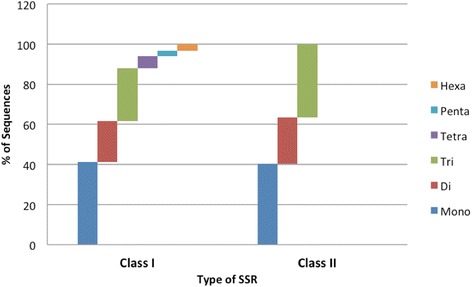
Distribution of Class I and Class II SSRs identified from *Spartina alterniflora* transcriptome. Class I SSRs are ≥ 20 bp while class II SSRs are ≥ 10 bp but < 20 bp in length

Scanning of all 273,460 unigenes representing 105.5 Mbp of the *S. alterniflora* genome for the presence of SSRs using MIcroSAtellite (MISA) Perl Script [72] showed that 9457 (3.45 %) unigenes contained at least one SSR and 920 (0.33 %) unigenes contained more than one SSR. The identified SSRs were either perfect (containing single motif repeat) or compound (contains two or more SSRs separated by ≤ 100 bp). Altogether, 10,805 SSRs were identified of which 1132 were compound type. Of the total 10,805 SSRs, 4895 (45.3 %) were mononucleotide repeats, 2237 (20.7 %) dinucleotide repeats, 3504 (32.4 %) trinucleotide repeats, 90 (0.8 %) tetranucleotide repeats, 38 (0.4 %) pentanucleotide repeats and 41 (0.4 %) were hexanucleotide repeats (Table 5). A total of 1185 (12.5 %) unigenes contained Class I SSRs (motif length of ≥ 20 bp) and 7666 (81.1 %) contained Class II SSRs (motif length ≥ 10 bp but < 20 bp). Trinucleotide repeats were 26.6 % in Class I as compared to 36.7 % in Class II SSRs. Tetra-, penta- and hexa-nucleotide repeats were least abundant in Class I SSRs and were not present in Class II SSRs (Fig. 11). Most of the SSRs were in the range of 5 to 15 repeat unit sizes, and the tri-nucleotide SSRs were the most abundant (Additional file 2: Figure S5). The average frequency of the SSRs in *S. alterniflora* transcriptome was found to be one per 9.76 kb. The AG/CT (52.3 %) and CCG/CGG (31.6 %) were the most abundant di- and trinucleotide type repeats (Additional file 2: Figure S6). The high abundance of AG and AT dinucleotides and least abundance of CG dinucleotides in *S. alterniflora* unigenes (Additional file 2: Figure S6) was similar to the observation in rice [73, 74]. Similar to the present finding, rice, barley and wheat also had abundance of SSRs with CCG trinucleotide motifs [73].

**Table 5:** Analysis of microsatellite markers in *Spartina alterniflora* unigenes

	LC	LS	RC	RS	Total
Total no. of sequences analyzed	73,552	68,346	72,199	59,363	273,460
Total size of sequences analyzed (Mbp)	30.34	30.29	23.82	21.02	105.48
Number of SSR containing sequences	3209	3188	1818	1242	9457(3.45 %)
Total number of identified SSR	3685	3654	2115	1351	10805
Number of sequences containing more than 1 SSR	334	343	175	68	920
Frequency of SSR found					1/9.76 kb
Dinucleotide SSR	599	668	588	382	2237 (20.7 %)
Trinucleotide SSR	1378	1341	496	289	3504(32.42 %)
Tetranucleotide SSR	13	25	43	9	90(0.83 %)
Pentanucleotide SSR	8	12	8	10	38(0.35 %)
Hexanucleotide SSR	12	10	8	11	41(0.37 %)

A total 3637 pairs of SSR primers were designed from the unigenes containing simple SSR motifs. Sequences, annealing temperatures and expected amplicon sizes of all the primers are provided in Additional file 4: Table S2. Location of SSRs could be assigned to 1729 SSR containing unigenes. Among them, 975 (56.4 %) were located in the protein coding region, 415 (24 %) were in the 5′-UTR and 339 (19.6 %) were in the 3′-UTR region. Of the 1,729 SSR containing unigenes, primer 3 could design SSR-flanking primers from only 1,338 unigenes (Additional file 4: Table S2). All genic SSRs tested were able to produce amplification products from all thirteen accessions (CP1 through CP 13) of *S. alterniflora* (Additional file 2: Figure S7). The average number of alleles per primer was 3.8 with a range of 3-6. Further, some of the SSRs produced accession-specific banding pattern. Large number of transcript-derived SSRs (ESSRs) identified in this study will enrich the genomic resources available for *S. alterniflora* [27, 75–77] for their use in variety identification, population genetic analysis, marker-trait analysis, and comparative genomics analysis because of their cross transferability etc.

## Conclusions

The present study is of significance for plant scientists involved in understanding salt stress responses in halophytes vis-à-vis glycophytes, because this is the first study which explores the *S. alterniflora* leaf and root transcriptome on a genome-wide scale to identify the candidate genes that are regulated by salt stress. The sequence information obtained from this research will add to the available resources in *Spartina* species for studying abiotic stress responses in non-model plants. Further, engineering of plants for incorporating halophyte traits based on the genes identified can produce salt tolerant cultivars. High similarity of *S. alterniflora* genes with rice genes makes *S. alterniflora* a good halophyte model for engineering of rice for salt tolerance, assuming that ectopic expression of orthologous genes from *S. alterniflora* perform similar function in rice. The hypothesis is that *S. alterniflora* maintains the state of stress anticipatory preparedness in non-stressful conditions, and operates more specific gene regulation and possesses unique adaptation machinery(ies) under high and prolonged salinity. The higher frequency of genes of unknown function (hypothetical genes) upregulated under salinity provided further clues to the involvement of novel genes or processes in *S. alterniflora* for adaptation to extreme environment. Further, enhanced salt tolerance of rice engineered with *S. alterniflora* genes provides stronger evidence that it is rational to use *S. alterniflora*, being a grass species, as a model resource as proposed by Flowers and Colmer (2008) for understanding the evolution of halophytic adaptation as well as for subsequent translation of the information to improving salt stress tolerance of cereal crops including rice, a major food crop of global importance. The large number of SSRs reported in this study will be useful for genetic diversity studies in *S. alterniflora*. Because of their cross-species transferability, the transcript-derived SSRs would facilitate genetic studies of different *Spartina* species for comparative genome analysis and evolutionary studies. Moreover, the SSRs derived from transcripts implicated in abiotic stress tolerances will be useful in identifying genotypes with stress tolerance alleles and candidate gene mapping.

## Additional files

Additional file 1: The comparative analysis among *Spartina alterniflora* reads with reads of *Salterniflora* [34] and unigenes of *S. pectinata* [78]. (XLSX 154 kb) Additional file 2: Figure S1. Percentage distribution of GC content between *Spartina alterniflora* and rice genes. **Figure S2** Gene family distribution among the four monocots, *Spartina alterniflora* (Sa), *Oryza sativa* (Os), *Sorghum bicolor* (Sb) and *Zea mays* (Zm). The homologous genes from each monocot species were clustered to represent gene family. The number of homologous genes shared by different species is represented by gene families at intersection. **Figure S3** Functional GO terms for gene families specific to *Spartina alterniflora* indicating coverage of different functional category genes specific to *S. alterniflora*. **Figure S4** A histogram showing the GC content distribution in different sets of genes of *Spartina alterniflora*. NP, set of genes having similarity outside of poaceae; PS, poaceae specific genes; All, whole *S. alterniflora* transcriptome; and SS, *S. alterniflora*-specific genes. **Figure S5** Distribution of different repeat unit size of the SSRs identified in *Spartina alterniflora* transcriptome. **Figure S6** Distribution of different types of SSR motifs in *Spartina alterniflora* unigenes. **Figure S7** Representative gel showing DNA profile of 13 (CP1 through CP13) *Spartina alterniflora* accessions produced by five SSR primers derived from the contigs. (PPTX 778 kb) Additional file 3: Table S1. Protein families found in *Spartina alterniflora* unigenes retrieved from Pfam database. (XLSX 229 kb) Additional file 4: Table S2. Simple sequence repeats (SSRs), flanking primers and their predicted location in *Spartina alterniflora* unigenes. (XLSX 357 kb)

## Abbreviations

DEG: Differentially expressed gene
FDR: False discovery rate
GO: Gene ontology
LC: Leaf control
LS: Stressed leaf
nr: Non-redundant
RC: Root control
RS: Stressed root
SSR: Simple sequence repeat
TF: Transcription factor
TR: Transcription regulator
