# Supplementary material for: Transcriptome analysis of smooth cordgrass (Spartina alterniflora Loisel), a monocot halophyte, reveals candidate genes involved in its adaptation to salinity
Source: BMC Genomics. 2016 Aug 19;17:657. doi: 10.1186/s12864-016-3017-3 (PMC4992267; doi:10.1186/s12864-016-3017-3)
Supplement: Additional file 2: Figure S1. — Percentage distribution of GC content between Spartina alterniflora and rice genes. Figure S2 Gene family distribution among the four monocots, Spartina alterniflora (Sa), Oryza sativa (Os), Sorghum bicolor (Sb) and Zea mays (Zm). The homologous genes from each monocot species were clustered to represent gene family. The number of homologous genes shared by different species is represented by gene families at intersection. Figure S3 Functional GO terms for gene families specific to Spartina alterniflora indicating coverage of different functional category genes specific to S. alterniflora. Figure S4 A histogram showing the GC content distribution in different sets of genes of Spartina alterniflora. NP, set of genes having similarity outside of poaceae; PS, poaceae specific genes; All, whole S. alterniflora transcriptome; and SS, S. alterniflora-specific genes. Figure S5 Distribution of different repeat unit size of the SSRs identified in Spartina alterniflora transcriptome. Figure S6 Distribution of different types of SSR motifs in Spartina alterniflora unigenes. Figure S7 Representative gel showing DNA profile of 13 (CP1 through CP13) Spartina alterniflora accessions produced by five SSR primers derived from the contigs. (PPTX 778 kb) [file 12864_2016_3017_MOESM2_ESM.pptx]

## Slide 1
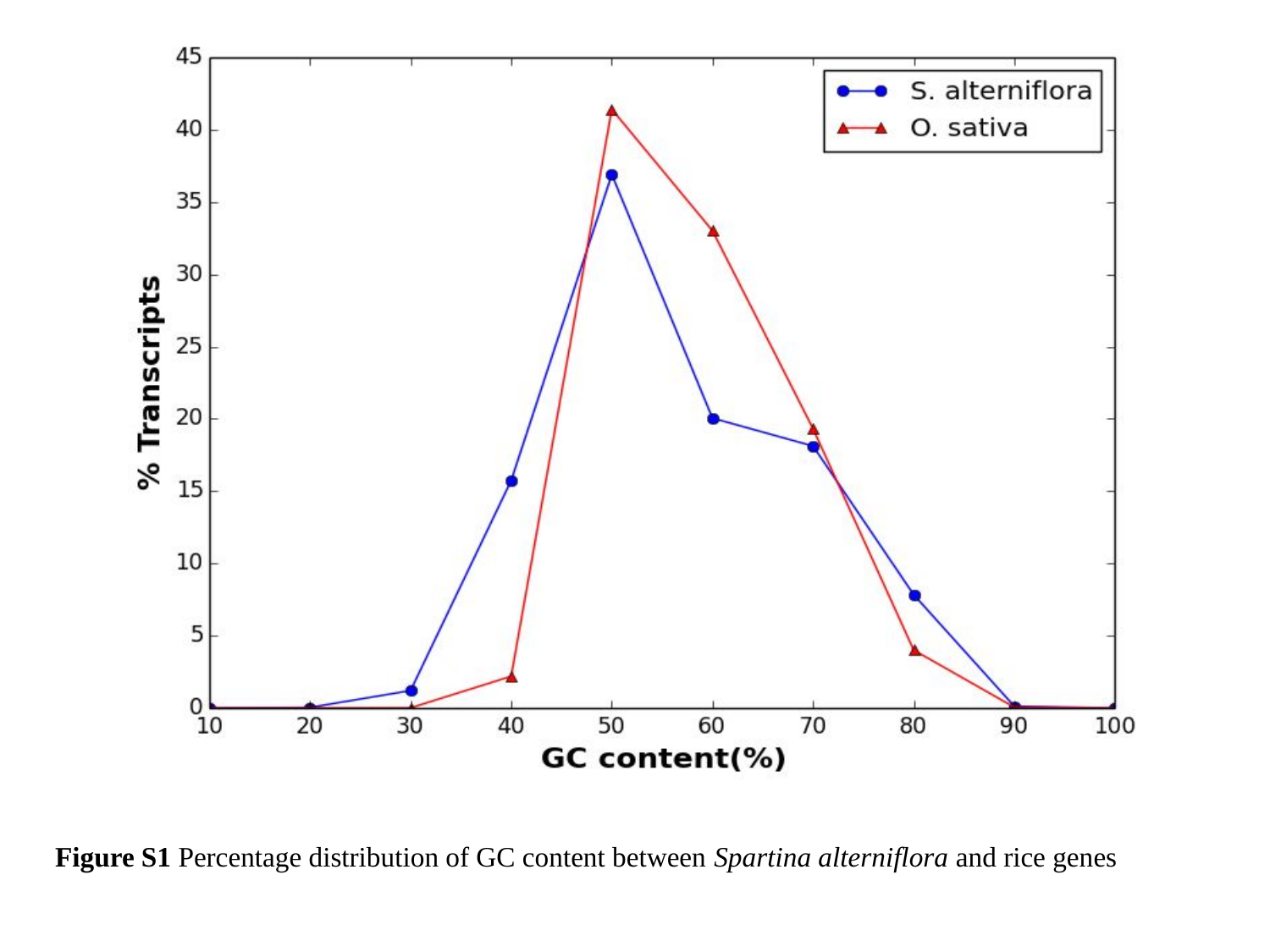

Figure S1 Percentage distribution of GC content between Spartina alterniflora and rice genes

## Slide 2
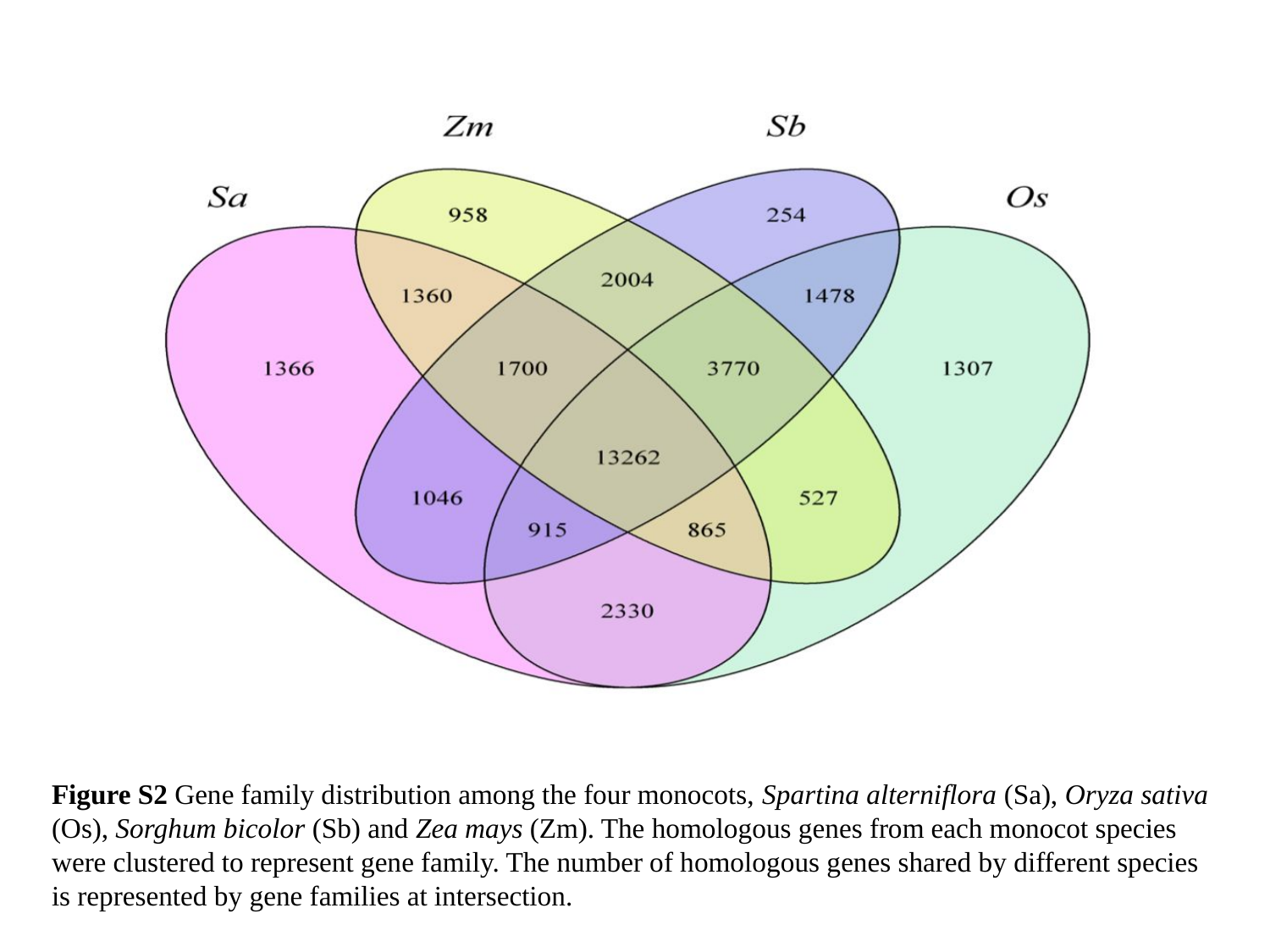

Figure S2 Gene family distribution among the four monocots, Spartina alterniflora (Sa), Oryza sativa (Os), Sorghum bicolor (Sb) and Zea mays (Zm). The homologous genes from each monocot species were clustered to represent gene family. The number of homologous genes shared by different species is represented by gene families at intersection.

## Slide 3
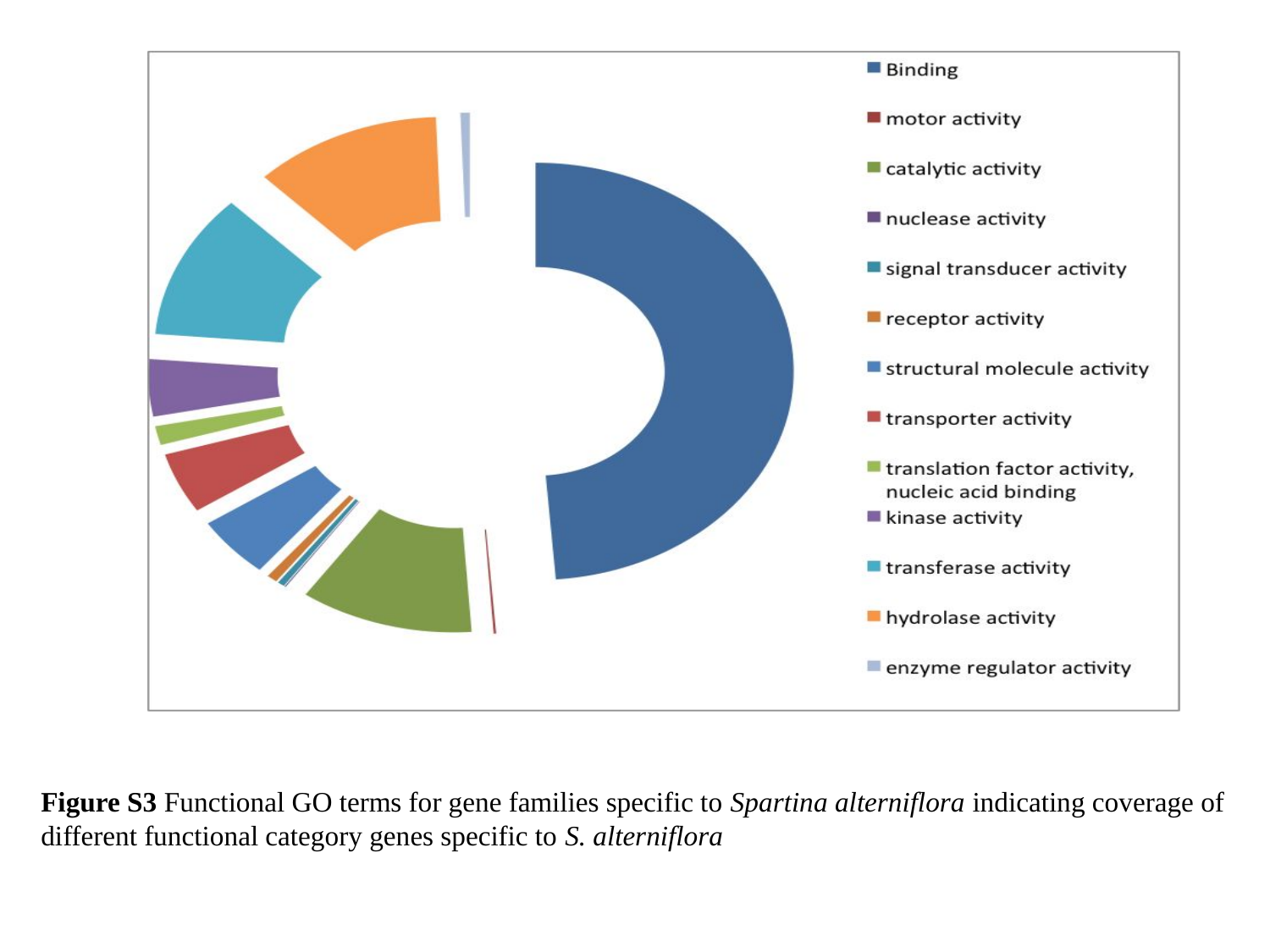

Figure S3 Functional GO terms for gene families specific to Spartina alterniflora indicating coverage of
different functional category genes specific to S. alterniflora

## Slide 4
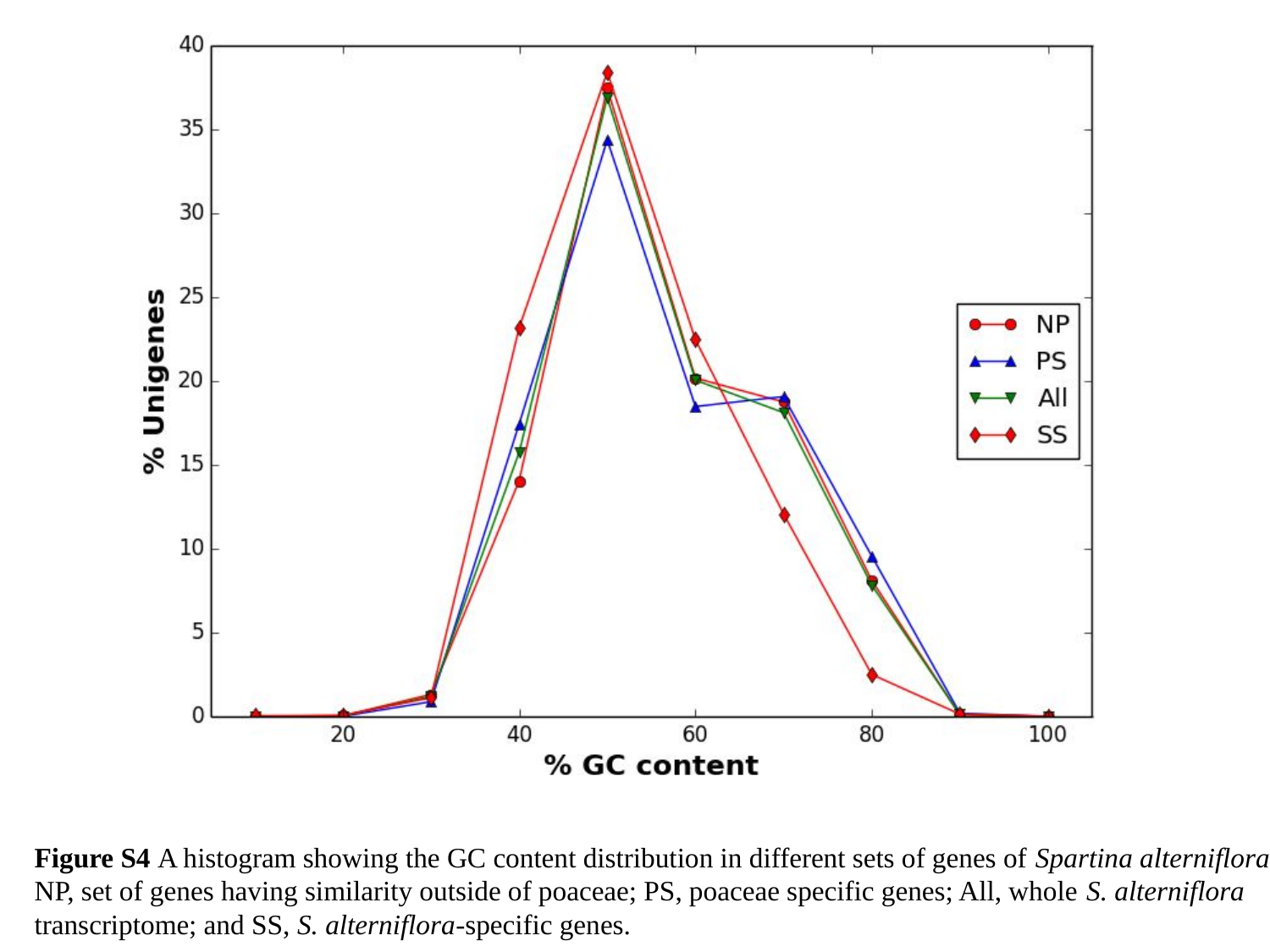

Figure S4 A histogram showing the GC content distribution in different sets of genes of Spartina alterniflora.
NP, set of genes having similarity outside of poaceae; PS, poaceae specific genes; All, whole S. alterniflora
transcriptome; and SS, S. alterniflora-specific genes.

## Slide 5
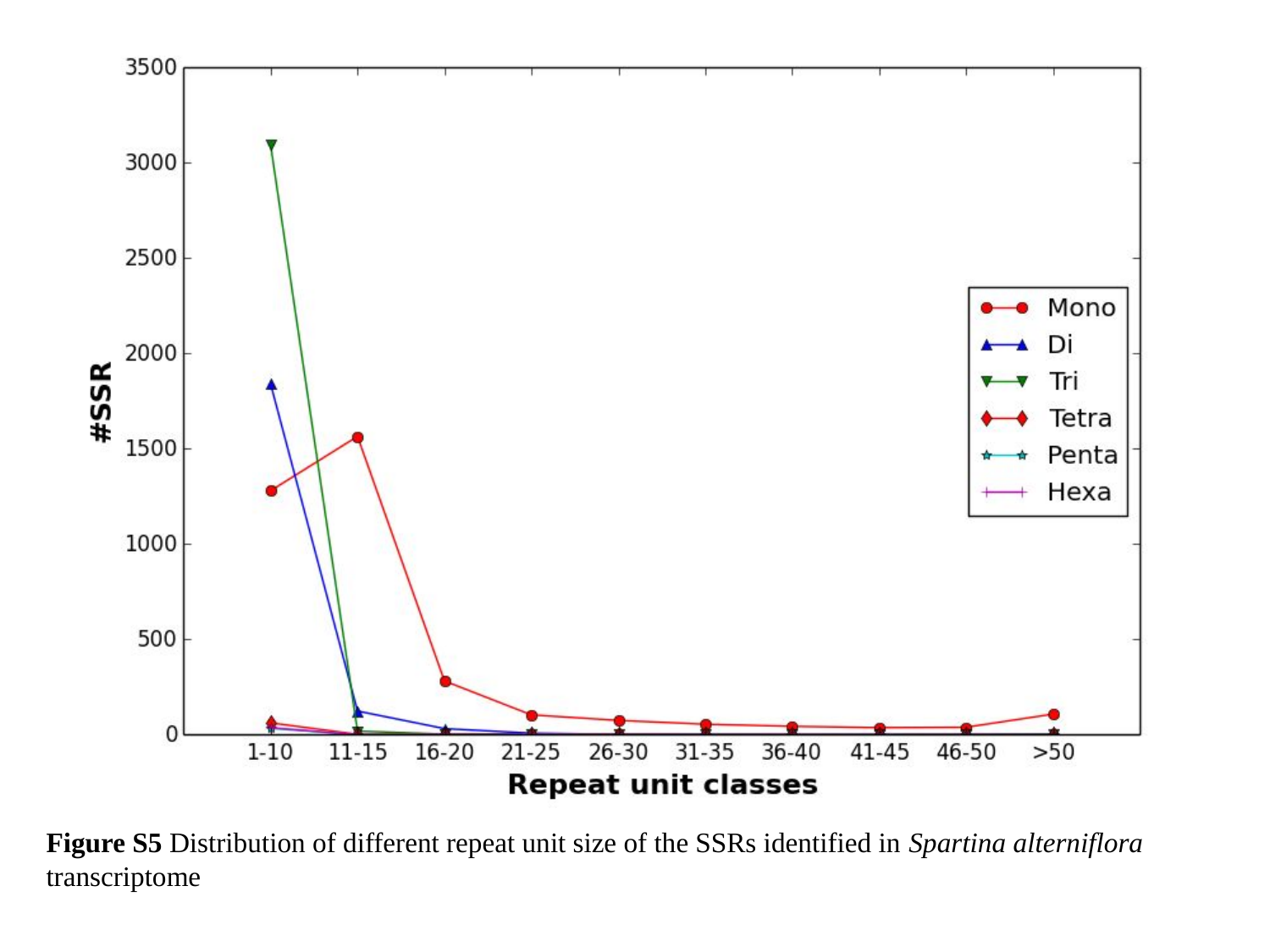

Figure S5 Distribution of different repeat unit size of the SSRs identified in Spartina alterniflora
transcriptome

## Slide 6
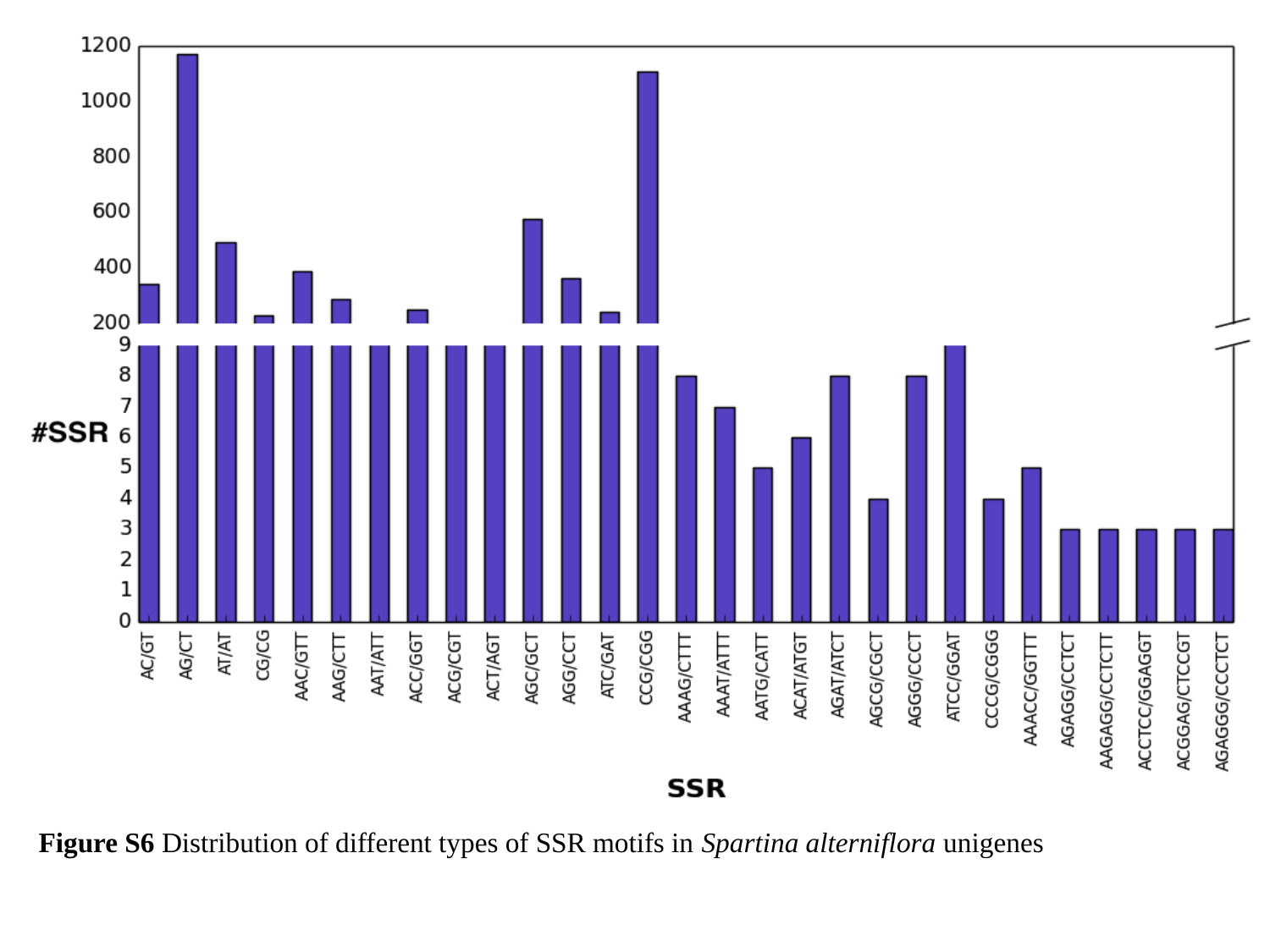

Figure S6 Distribution of different types of SSR motifs in Spartina alterniflora unigenes

## Slide 7
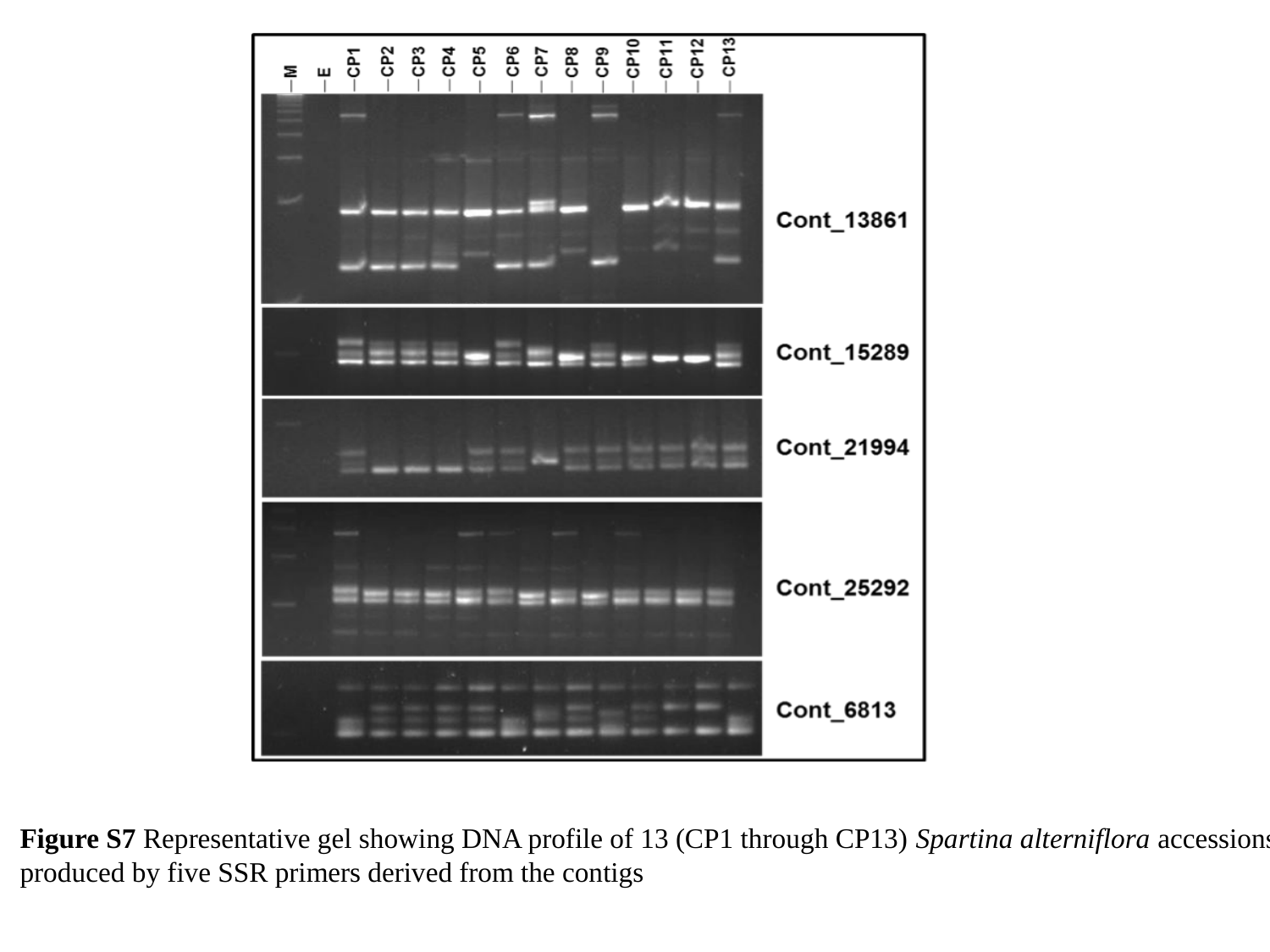

Figure S7 Representative gel showing DNA profile of 13 (CP1 through CP13) Spartina alterniflora accessions
produced by five SSR primers derived from the contigs
